# Microtubules and Their Role in Cellular Stress in Cancer

**DOI:** 10.3389/fonc.2014.00153

**Published:** 2014-06-18

**Authors:** Amelia L. Parker, Maria Kavallaris, Joshua A. McCarroll

**Affiliations:** ^1^Tumour Biology and Targeting Program, Children’s Cancer Institute Australia, Lowy Cancer Research Centre, University of New South Wales, Sydney, NSW, Australia; ^2^Australian Centre for NanoMedicine, University of New South Wales, Sydney, NSW, Australia

**Keywords:** microtubules, tubulin, post-translational modifications, microtubule-associated proteins, stress response

## Abstract

Microtubules are highly dynamic structures, which consist of α- and β-tubulin heterodimers, and are involved in cell movement, intracellular trafficking, and mitosis. In the context of cancer, the tubulin family of proteins is recognized as the target of the tubulin-binding chemotherapeutics, which suppress the dynamics of the mitotic spindle to cause mitotic arrest and cell death. Importantly, changes in microtubule stability and the expression of different tubulin isotypes as well as altered post-translational modifications have been reported for a range of cancers. These changes have been correlated with poor prognosis and chemotherapy resistance in solid and hematological cancers. However, the mechanisms underlying these observations have remained poorly understood. Emerging evidence suggests that tubulins and microtubule-associated proteins may play a role in a range of cellular stress responses, thus conferring survival advantage to cancer cells. This review will focus on the importance of the microtubule–protein network in regulating critical cellular processes in response to stress. Understanding the role of microtubules in this context may offer novel therapeutic approaches for the treatment of cancer.

## Introduction

Microtubules, together with microfilaments and intermediate filaments, form the cell cytoskeleton. The microtubule network is recognized for its role in regulating cell growth and movement as well as key signaling events, which modulate fundamental cellular processes. Emerging evidence also suggests that it is critically involved in cell stress responses. This review will focus on the role of microtubules in this context in cancer.

**Figure 1:**
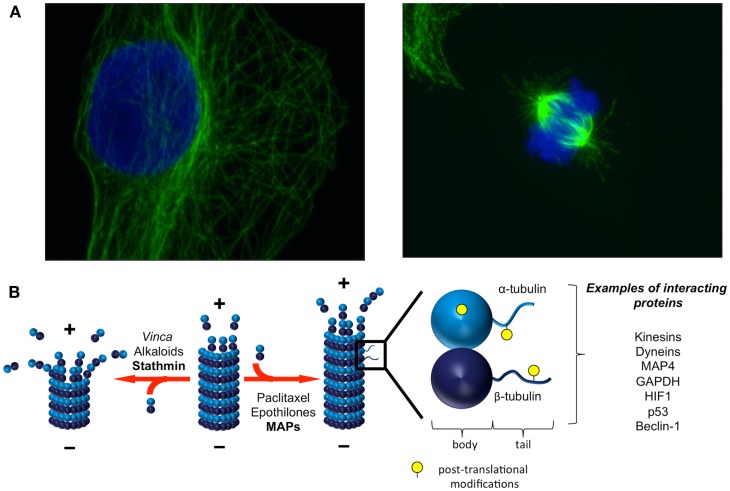
**Microtubules are dynamic structures that interact with diverse proteins**. **(A)** Microtubules form a dynamic network and are constantly lengthening and shortening. In interphase [**(A)**, left], microtubules are anchored at the centrosome (minus end) and radiate toward the cell periphery (plus end). The microtubule network undergoes dramatic remodeling throughout the cell cycle, from interphase and through mitosis [**(A)**, right]. Green: α-tubulin, blue: DAPI. Images courtesy of Dr. Sela Po’uha. **(B)** Heterodimers of α- and β-tubulin associate to form microtubules. The dynamic addition and removal of tubulin heterodimers is faster at microtubule plus ends than at microtubule minus ends. Both endogenous factors and TBAs regulate and influence microtubule dynamics. A variety of proteins involved in cellular homeostatic mechanisms and stress responses also interact with tubulins either in their soluble or polymerized forms. Post-translational modifications on tubulins influence these interactions. Adapted with permission from Macmillan Publishers Ltd: Nature Reviews Molecular Cell Biology [Ref. (9)], Copyright 2011 and Nature Reviews Cancer [Ref. (15)], Copyright 2010.

Microtubules are composed of α- and β-tubulin heterodimers that associate to form hollow cylindrical structures (1) (Figure 1). They are highly dynamic, and are constantly lengthening and shortening throughout all phases of the cell cycle. During interphase, microtubules are nucleated at the centrosome (minus end) and radiate toward the cell periphery (plus end). Interphase microtubules are involved in the maintenance of cell shape and in the trafficking of proteins and organelles (1). Motor proteins translocate cell components on microtubule tracks, and protein–protein interactions with other adaptor proteins co-ordinate this process. Tubulin heterodimers also exist in soluble form in cells, and protein interactions with this tubulin population regulate microtubule behavior.

The addition and removal of soluble tubulin heterodimers to dynamic microtubule ends is a highly regulated process (Figure 1). Tubulin dimers are nucleotide binding proteins, with β-tubulin also possessing GTPase activity. The manner in which tubulin heterodimers are orientated in microtubules gives rise to a polar molecule that differs in both structure and kinetics at each end of the microtubule. The dynamics of tubulin addition and release are much slower at the minus end of the microtubule, which terminates with α-tubulin proteins, compared with the plus end of the microtubule, which terminates with β-tubulin proteins. The addition of a tubulin heterodimer to a microtubule activates the GTPase activity of β-tubulin, locking the β-tubulins in the microtubule in a GDP-bound state. The β-tubulins exposed to the solvent at the end of the microtubule form a GTP cap that is important in preventing microtubule depolymerization. Therefore, the binding of GTP at the microtubule plus end imparts structural and kinetic polarity to microtubules and is an important regulator of microtubule stability. It is believed that the polymerized and soluble tubulin pools interact with different signaling networks, however, the dynamic exchange of tubulin subunits between these pools makes it difficult to distinguish the functional roles of soluble and polymerized tubulin experimentally. The reader is referred to several excellent reviews for more detailed information on microtubule structure and dynamics (1, 2).

During mitosis, microtubules form the spindle to enable correct chromosomal segregation (3). Tubulin-binding agents (TBAs; e.g., taxanes, vinca alkaloids, epothilones, and eribulin) are important chemotherapeutic drugs that suppress spindle dynamics, causing subsequent mitotic arrest and cell death in rapidly dividing cells (3). Recent evidence suggests that the induction of cell stress in interphase cells also contributes significantly to TBA-mediated cell death (4–6), highlighting the importance of tubulin in cell stress responses in cancer.

In humans, microtubules are composed of combinations of eight α-tubulin isotypes and seven β-tubulin isotypes, with the different tubulin isotypes possessing specific tissue and developmental distributions (7) (Table 1). The members of the tubulin family share a high degree of structural homology and are distinguished from one another by highly divergent sequences at their carboxy-terminal (C-terminal) tail (8). The C-terminal tails of tubulin are also thought to mediate protein–protein interactions and act as sites of post-translational modifications to confer unique functionality to each isotype (9).

**Table 1:** **Tubulin isotypes present in humans [Adapted with permission from Macmillan Publishers Ltd: Nature Reviews Cancer (Ref. (15)) Copyright 2010 and Elsevier (Ref. (233)) Copyright 2009]**.

Tubulin isotype	Gene name	Accession number
**α-TUBULIN**		
α1A-Tubulin	TUBA1A	NP_006000
α1B-Tubulin	TUBA1B	AAC31959
α1C-Tubulin	TUBA1C	Q9BQE3
α3C-Tubulin	TUBA3C	Q13748
α3E-Tubulin	TUBA3E	NP_997195
α4A-Tubulin	TUBA4A	NP_005991
α8-Tubulin	TUBA8	Q9NY65
α-Like 3-Tubulin	TUBAL3	NP_079079
**β-TUBULIN**		
βI-Tubulin	TUBB	NM_178014
βII-Tubulin	TUBB2A, TUBB2B	NM_001069; NM_178012
βIII-Tubulin	TUBB3	NM_006086
βIVa-Tubulin	TUBB4	NM_006087
βIVb-Tubulin	TUBB2C	NM_006088
βV-Tubulin	TUBB6	NM_032525
βVI-Tubulin	TUBB1	NM_030773

*The authors direct readers to comprehensive reviews (233) for further information on tubulin isotype structure*.

## Tubulin Alterations in Cancer

Diverse changes in the microtubule network have been identified and characterized in a wide variety of cancers, including altered expression of tubulin isotypes, alterations in tubulin post-translational modifications, and changes in the expression of microtubule-associated proteins (MAPs) (Table 2). Despite evidence from *in vitro* studies associating tubulin mutations with resistance to TBAs (10–13), tubulin mutations are not clinically prevalent and their importance in disease progression and chemotherapy resistance is controversial (14). Microtubule alterations are thought to influence cellular responses to chemotherapeutic and microenvironmental stressors, thereby contributing to broad spectrum chemotherapy resistance, tumor development, and cell survival.

**Table 2:** **Clinical studies of tubulin alterations in cancer**.

Microtubule alteration	Observation	Effect	Cancer	Reference
Altered isotype expression	High βI-tubulin	Poor response to docetaxel treatment	Breast cancer	(234)
	High βIII-tubulin expression	Poor survival, poor outcome for surgical resection or TBA response; correlates with subtype	Non-small cell lung cancer (NSCLC)	(21, 31, 108, 235–238)
		Correlates with poor survival, poor response to platinum and taxane treatment, advanced stage, or aggressive disease	Ovarian cancer	(13, 16, 35, 239–242)
		Favorable response to taxane treatment	Ovarian (clear cell adenocarcinoma)	(243)
		Poor response to taxane treatment	Breast cancer	(234, 244)
		Correlates with disease stage	Pancreatic ductal adenocarcinoma	(17)
		Correlates with disease stage	Glioblastoma	(101)
		Localized to invasive edge	Colorectal cancer	(245)
		Poor response to taxane/platinum treatment	Uterine serous carcinoma	(246)
		Poor response to taxane treatment	Gastric cancer	(247)
		Aggressive disease, patient outcome	Prostate cancer	(36, 248, 249)
	Low βII-tubulin expression	Correlates with poor response to taxane treatment or advanced stage disease	Breast and ovarian cancer	(239, 250)
	High βIVa-tubulin expression	Poor response to taxol treatment	Ovarian cancer	(240)
	High βV-tubulin expression	Favorable response to taxane treatment	NSCLC	(251)
	High α1b-tubulin expression	Histological grade	Hepatocellular carcinoma	(252)
	High γ-tubulin expression	Poorly differentiated	Medulloblastoma	(253)
Altered post-translational modification	High Δ2α-tubulin	Poor response to vinca alkaloid treatment	Advanced NSCLC	(238)
	High detyrosinated tubulin	Disease aggressiveness	Breast cancer	(48)
	Active tyrosination cycle	Favorable patient outcome	Neuroblastoma	(50)

### Changes in tubulin isotype composition

Altered tubulin isotype expression is the most widely characterized microtubule alteration reported in cancer and has been observed in both solid and hematological tumors. These changes are often associated with chemotherapy resistance and poor prognosis (Table 2) [reviewed in Ref. (15)]. Compared with α-tubulin isotypes, β-tubulin isotypes have received more attention in this context, largely due to the availability of isotype-specific antibodies, and the fact that TBAs bind to the β-tubulin subunit to exert their toxic effect. Furthermore, βIII-tubulin is the most comprehensively examined isotype across a variety of cancers.

Elevated βIII-tubulin levels are associated with poor prognosis in a host of different epithelial cancers. In addition to TBA resistance, βIII-tubulin levels influence sensitivity to non-tubulin-targeted agents [reviewed in Ref. (15)]. The clinical observations are supported by numerous *in vitro* studies where altered βIII-tubulin levels confer resistance to a broad spectrum of drug classes in solid and hematological tumors [reviewed in Ref. (15)]. Coupled with evidence that βIII-tubulin is also involved in tumor development and disease aggressiveness (16–18), these results suggest that βIII-tubulin may be acting as a survival factor in cancer.

Altered levels of βII-, βIVa-, βIVb-, and βV-tubulins have also been associated with resistance to TBAs in a number of drug resistant cancer cell types (19–26). However, the clinical relevance of these specific tubulin isotypes is limited and requires further investigation. Moreover, the involvement of tubulin isotypes in disease progression is complex, and depends on both the treatment regime and disease stage (27). Additional complexity may be conferred by interactions between different isotypes, since the overexpression of specific β-tubulin isotypes, such as βI, βII, and βIVb, does not affect TBA resistance in Chinese Hamster Ovary cells (28, 29). For βIII-tubulin the results have been conflicting. Overexpression of βIII-tubulin failed to confer resistance to TBAs in prostate cancer (28, 29). In contrast, overexpressing this isotype in Chinese Hamster Ovary cells conferred resistance to paclitaxel (30).

In cancer, alterations in the tubulin isotype composition have been detected at both the gene and protein level and result from increased gene transcription and enhanced mRNA stability (24). However, tubulin mRNA levels do not always reflect protein expression due to the complexity of post-translational mechanisms that control tubulin expression (24, 31). For instance, the tumor suppressor miR-100 and the miR-200 family of microRNAs (24, 32, 33) as well as epigenetic mechanisms (34, 35) are implicated in coordinating β-tubulin isotype expression. Therefore, dysregulation of miRNA networks and epigenetic mechanisms in cancer may also contribute to aberrant tubulin isotype expression in cancer. Recent evidence showing an association between elevated βIII-tubulin expression and PTEN deletions in prostate cancer also suggest that changes in the levels of this isotype may result from PTEN-mediated genetic reprograming (36).

Cell stress influences the tubulin isotype composition. For example, βIII-tubulin expression can be induced (24, 37) or decreased (16) by chemotherapy treatment. The induction of βIII-tubulin has been observed in response to vinca alkaloid treatment in breast cancer cells through an activator protein-1 (AP-1) site on the βIII-tubulin promoter (38), while its induction in hypoxic and hypoglycemic conditions in ovarian cancer cells is mediated by hypoxia-inducible factor (HIF) 1α and Hu antigen (HuR), respectively, at the 3′ untranslated region (UTR) (39, 40). The latter mechanism is a regulatory feature commonly utilized by proteins involved in cell stress, and enables rapid changes in protein levels (41). However, it is to be noted that the regulation of βIII-tubulin levels in cell stress responses may depend upon the basal expression of the protein and may also be cell type specific.

Initially, differences in the drug binding affinity and structural characteristics of microtubules composed of different β-tubulin isotypes were thought to explain correlations between aberrant tubulin isotype compositions and resistance to TBAs. However, recent observations correlating changes in isotype expression with tumor development and resistance to non-TBA agents have challenged the simplicity of this model. With increased recognition of the importance of cell stress responses in chemotherapy efficacy, isotype-mediated modulation of these responses may contribute to chemotherapy resistance. In particular, cellular homeostasis relies on a dynamic microtubule network and may be perturbed by alterations in microtubule stability and dynamics. The microtubule isotype composition does affect microtubule stability, with consequences for TBA sensitivity (7, 23, 42). Stable microtubules play an important role in cellular trafficking and their role in multiple stress responses are discussed below. Chemotherapy agents that do not bind to tubulin can also affect microtubule stability by unknown mechanisms (43), and this may represent a mechanism common to chemotherapy agents of different classes.

The tubulin isotype composition can also influence microtubule dynamics. In non-small cell lung cancer (NSCLC) cells, suppression of βIII-tubulin using RNA interference technology decreases microtubule dynamics in the presence of TBAs, but has no effect under basal conditions (44). These observations suggest that changes in isotype composition may influence microtubule dynamics in the presence of chemotherapeutic stressors but not under basal conditions; however, a direct causal relationship between isotype expression, microtubule dynamics, and cell survival in response to these and other stressors has not been established. In general, the importance of microtubule dynamics in homeostatic cell signaling suggests that cell stress responses, and not just spindle dynamics, may be impacted by aberrant isotype expression in cancer, thus offering an additional determinant of chemosensitivity.

### Tubulin post-translational modifications

Tubulins are subject to diverse post-translational modifications (PTMs) [reviewed in Ref. (9)]. The majority of tubulin PTMs are highly heterogeneous, and little is understood about the regulation and impact of these modifications. Post-translational modifications are thought to regulate protein–protein interactions with the microtubule cytoskeleton, thereby affecting signaling events within the cell. The majority of these modifications are localized to the tubulin C-terminus and potentially impart specific functions to the different tubulin isotypes.

Removal and addition of the α-tubulin C-terminal tyrosine occurs cyclically in cells. Tyrosine addition and removal are catalyzed by tubulin tyrosine ligase (TTL), and carboxypeptidases, respectively (9). Highly dynamic microtubules are more likely to be detyrosinated, due to the kinetic balance between higher TTL and carboxypeptidase activities on the soluble and polymerized tubulin pools, respectively (45). While traditionally viewed as an intrinsic hallmark of stable microtubules, the detyrosination motif alters motor protein recruitment to microtubules, thereby stabilizing microtubules and influencing trafficking functions within the cell (46).

Tyrosination modifications of α-tubulin are known to be critical for differentiation, cell cycle progression, organelle trafficking, and vesicular transport (9). Altered levels of tyrosination modifications and the enzymes responsible for them have been detected in a range of cancers and are associated with more aggressive disease (47–50). For instance, loss of TTL induces mesenchymal transition in breast cancer cells, which may contribute to increased metastatic potential and altered cell stress responses (51).

Increased acetylation of α-tubulin on Lys40 has also been observed in tumor cells (52). Elevated HDAC6 expression, one of several regulators of tubulin acetylation, is associated with better prognosis in breast cancer (53). Sirtuin-2 is also responsible for tubulin deacetylation (54) and has been linked with the regulation of autophagy in response to stress [reviewed in Ref. (55)]. HDAC6 does influence microtubule stability (56), however, whether acetylation itself influences microtubule stability remains uncertain. Acetylated tubulin is implicated in intracellular trafficking (57), endoplasmic reticulum (ER) localization, and ER–mitochondria interactions (58), as well as the regulation of microtubule dynamics (59). The involvement of α-tubulin acetylation in a broad range of cell functions may reflect its importance in the maintenance of cellular homeostasis.

Other post-translational modifications have been detected in prostate and hepatic cancers. Removal of the final two residues of the βIVb-tubulin C-terminal tail was identified in higher stage liver cancer and in a mouse model of hepatic carcinoma (60). Polyglutamylated α-tubulins (47) and the polyglutamylation enzyme TTL-like 12 are elevated in prostate cancer and correlate with more aggressive disease (61).

Overall, despite a lack of clarity surrounding the mechanistic details of the function of tubulin PTMs, mounting evidence points to their role in fundamental cell processes. The diverse PTM alterations observed in a range of cancers are likely to perturb homeostatic processes, thereby contributing to stress response signaling. Detailed spatiotemporal mapping of tubulin PTMs and proteomic studies investigating their role in signaling networks are required to elucidate the influence of tubulin PTMs on cellular stress responses.

### Microtubule-associated proteins

A wide variety of proteins are known to interact with tubulins. Interactions between tubulin and MAPs influence microtubule stability and dynamics, and are known to affect chemotherapy sensitivity and tumor growth in cancer [reviewed in Ref. (62)]. Aberrant expression of primarily neuronal MAPs (e.g., Tau, MAP2) has been detected in non-neuronal cancer tissue. For example, tau overexpression is correlated with poor outcome in breast cancer, and this protein may influence taxane sensitivity by decreasing the affinity of the drug for β-tubulin (63). Altered MAP2 expression is also associated with taxane resistance (22, 64), with differential effects in primary and metastatic melanoma (65).

Increased MAP4 expression and altered expression of multiple MAP4 isoforms have been detected in TBA-resistant leukemia and NSCLC cells *in vitro* (10, 11, 66). In addition, changes in stathmin, survivin, BRCA1, CLIP170, and VHL expression have all been associated with chemotherapy resistance and disease progression (62, 67). For instance, stathmin was recently shown to play an important role in regulating neuroblastoma cell migration and invasion (68). Moreover, silencing its expression using RNAi gene-silencing technology significantly reduced lung metastases in a clinically relevant orthotopic neuroblastoma mouse model (68). The overexpression of kinesins also influences chemotherapy sensitivity and disease progression through mitotic and non-mitotic mechanisms [reviewed in Ref. (69)]. A recent study has shown that kinesins interact differentially and specifically with tubulin isotypes and tubulin post-translational modifications (70). In this way, changes in tubulin isotype expression and post-translational modifications seen in cancer may also influence motor protein function and the numerous basic processes that depend upon these interactions.

The effect of MAPs on cell function in cancer is complex, with interactions between individual MAPs influencing survival and metastases. Progress toward understanding the functional consequences of these proteins and their signaling networks in cancer relies upon more comprehensive characterization of the interactions between tubulins and MAPs, and the influence of tubulin isotypes and PTMs on these interactions.

## Microtubule Cytoskeleton in Stress Responses

Microtubules influence homeostatic mechanisms and cell stress responses by regulating intracellular trafficking, acting as a scaffold for the co-localization and sequestration of stress response proteins, transmitting stress signals through cytoskeletal remodeling and modulating the induction of cell death pathways. Examples of their role in these processes are described below.

### Microtubules and cellular signaling

While microtubules possess distinct functions in particular stress responses, the microtubule network also influences common signaling pathways engaged by a variety of cellular stresses. Stress response signaling requires trafficking of proteins and organelles throughout the cell and modulation of the microtubule network is expected to influence signal transduction events. For example, TBAs differentially suppress microtubule-mediated intracellular transport in neuronal cells (71).

In addition to general effects on signal transduction, microtubules regulate mitogen activated protein kinase (MAPK) signaling. The MAPK superfamily includes extracellular regulated kinases (ERK), c-Jun N-terminal protein kinase (JNK), and p38 families and is critically involved in mediating the initiation and execution of a range of cellular stress responses [reviewed in Ref. (72)]. MAPK proteins interact extensively with the microtubule network, with one-third of the total MAPKs associating with microtubules through kinesin motor proteins (73). Interactions between microtubules and these signaling proteins can regulate and co-ordinate widespread cellular stress signaling events.

The JNK signaling pathway is induced by a wide range of environmental stressors (72) and TBAs activate this pathway in the induction of apoptosis (74–76). In particular, JNK signaling is required for the execution of apoptosis in response to ER stress and autophagy (77). JNK co-ordinates cytoskeletal architecture in normal cells and JNK1 regulates microtubule dynamics (78, 79). JNK1 also phosphorylates MAP1 and MAP2 to alter their distribution and microtubule architecture (79). In this context, JNK, the heavy chain kinesin family-5B protein and βIII-tubulin form a complex, raising the possibility that alterations in β-tubulin isotype composition may affect JNK pathway activation and cell death responses.

While TBAs generally activate JNK signaling to initiate apoptosis [reviewed in Ref. (80)], microtubule stabilizing and destabilizing agents differentially influence downstream signaling events, suggesting that microtubule stability regulates JNK signaling (81). Compared with etoposide and doxorubicin, vinblastine uniquely causes c-Jun phosphorylation, AP-1 activation, ERK inactivation, and p53 downregulation (81). Microtubule destabilizing and stabilizing agents initiate apoptosis via JNK signaling through AP-1 dependent and AP-1 independent mechanisms, respectively (82). The AP-1 dependent pathway leads to positive feedback of c-Jun levels and sustained JNK signaling (82), suggesting that microtubule–JNK interactions may constitute a feedback loop for the amplification and damping of signaling pathways to regulate stress response kinetics.

Extracellular regulated kinase also interacts with microtubules and phosphorylates MAPs to regulate their activity (83, 84). MAPK-mediated MAP phosphorylation is implicated in hypoxic stress responses (85). Differential induction of ERK signaling by TBAs may also mediate downstream effects independently of apoptosis induction (86).

It is well established that microtubules are involved in the translocation of messenger proteins between different cell compartments to enable efficient signal transduction. However, increasing evidence supports a role for microtubule dynamics, tubulin isotypes, and MAPs in specifically regulating the course, amplitude, and kinetics of MAPK signaling.

### p53 and microtubules

p53 is a key mediator of cellular stress responses and its activity heavily depends on microtubules (87). p53 is translocated to the nucleus along microtubule tracks by dynein proteins in a complex with heat shock protein 90 (Hsp90) and Hsp90 immunophilins (87–89). The binding of Hsp90 to p53 inhibits MDM2-mediated degradation of the protein by the ubiquitin–proteasome system (90).

Microtubule dynamics regulate p53 levels. p53 levels and its nuclear accumulation are increased by TBA treatment at doses that suppress microtubule dynamics but do not disrupt the structure of the microtubule network (87, 91). MAP1B also associates with p53, decreasing its activity and inhibiting doxorubicin-induced apoptosis in neuroblastoma cells (92). p53 signaling can influence microtubule dynamics and remodeling, as well as the expression of tubulin isotypes and MAPs (93). Taken together, by regulating p53 levels and translocation, microtubules significantly impact p53-mediated stress response signaling.

### Hypoxia

Rapid cell proliferation and poor vascular development leads to hypoxic regions within solid tumors. Hypoxia-inducible factor 1 (HIF1) is considered to be the master regulator of cellular adaptation to hypoxia and is upregulated in a large proportion of solid cancers (94).

In the absence of oxygen, HIF1α heterodimerizes to the constitutively active β subunit to initiate transcriptional changes [reviewed in Ref. (95)]. HIF1α stabilization is regulated by enzyme-mediated hydroxylation, which enables recognition of HIF1α for ubiquitinylation and degradation by proteins such as the von Hippel–Lindau (VHL) protein (96). Low oxygen levels inactivate the hydroxylases, leading to stabilization and nuclear translocation of the α subunit where the HIF1 heterodimer binds to hypoxia responsive elements in target gene promoters (95).

Dramatic microtubule remodeling occurs under hypoxic conditions. Decreased microtubule polymerization has been observed in response to anoxic conditions (0–2% O_2_) (85, 97), while increased microtubule polymerization has been observed in physiological hypoxia (3% O_2_) (98). Enhanced microtubule polymerization under these conditions is coupled with increased tubulin detyrosination and glycogen synthase kinase 3β (GSK3β) inhibition (98), while phosphorylation of the MAPs dynein light chain tctex-type 1 (DYNLT1), MAP4, and stathmin have each been associated with microtubule depolymerization (85). Discrepancies between these observations may be due to the differential effects of anoxia compared with physiological hypoxia, or alternatively may reflect the role of the GSK3β pathway and MAP interactions on microtubule remodeling (98). Hypoxic activation of the p38/MAPK pathway contributes to phosphorylation of MAP4 and stathmin (85). Microtubule remodeling in response to hypoxia may impact metastatic processes with increased microtubule polymerization influencing integrin trafficking and invasion in breast cancer cells (98).

MAP4 protects against microtubule disruption during hypoxia by enhancing tubulin polymerization and concomitant upregulation of tubulin expression (97). It also maintains ATP production under hypoxic conditions and prevents mitochondrial permeabilization (97). The non-phosphorylated form of DYNLT1 also protects against microtubule disruption and mitochondrial permeabilization and maintains the cellular energy status in hypoxia, with phosphorylation of DYNLT1 potentiating cell death through mitochondrial permeabilization (99). DYNLT1-mediated interactions between tubulin and Voltage Dependent Anion Channels (VDACs) may facilitate cross-talk between the microtubule cytoskeleton, intrinsic apoptotic pathway, and mitochondrial quality control system to influence cell survival in hypoxia (97).

Hypoxic adaptation may also be regulated by specific tubulin isotypes in cancer cells. For instance, βIII-tubulin (encoded by the TUBB3 gene) is induced under hypoxic conditions by direct binding of HIF1α to the E box motif within its 3′UTR (39). Hypoxic upregulation of this isotype appears to be cell type specific, depends on the epigenetic status of the TUBB3 3′UTR and is also influenced by the basal βIII-tubulin expression level (26, 39). The expression of this tubulin isotype is also regulated by HuR (40), which is involved in HIF1α stabilization (100). High βIII-tubulin expression is also detected in close proximity to necrotic tumor regions, further supporting a role for this protein in hypoxic adaptation (101).

Hypoxia-inducible factor 1α degradation is dependent on the short isoform of VHL, while the long isoform is a known regulator of microtubule dynamics (102). In renal cell carcinoma, where VHL mutations result in upregulated HIF expression, there is a loss of microtubule–HIF coupling, suggesting that VHL may be responsible for microtubule-mediated regulation of HIF signaling (103). However, the mechanisms underlying this observation and the functional consequences of this regulatory process are uncertain.

Hypoxia-inducible factor 1 activity depends upon its ability to translocate to the nucleus, and microtubules act as tracks for dynein-mediated HIF1 translocation (103). Suppression of microtubule dynamics decreases HIF1α levels by increasing HIF1α mRNA association with inactive ribosomal subunits and by targeting this mRNA to P-body components (104). Suppression of microtubule dynamics and HIF nuclear translocation prevents VEGF-mediated hypoxic adaptation in prostate and breast cancer cells and decreases angiogenesis in a murine orthotopic breast tumor model (94). However in this study, microtubule dynamics regulated HIF1α levels to the same extent in both normoxic and hypoxic conditions; therefore, this mechanism may not be responsible for regulating HIF1α levels specifically in response to hypoxia. Recent evidence suggests that hypoxic adaptation also depends upon microtubule-mediated perinuclear mitochondrial clustering (105), and highlights the importance of organelle localization in cellular adaptation to hypoxia.

Overall, the hypoxic response is associated with dramatic microtubule remodeling, and altered MAP signaling to maintain bioenergetics and organelle function under hypoxic conditions. The microtubule network also regulates hypoxic adaptation by affecting HIF1α signaling and organelle localization, placing microtubules as a central player in the hypoxic stress response. While current evidence suggests that β-tubulins may function in an isotype-specific manner in this context, a more comprehensive analysis of the contributions of each individual isotype to hypoxic adaptation is required.

### Oxidative stress

Aberrant oxidative stress signaling has been reported in many cancers. The upregulation of enzymes responsible for redox homeostasis, metabolic reprograming, and exposure to extracellular inducers of intracellular oxidative species all contribute to aberrant oxidative conditions in cancer [reviewed in Ref. (106)]. Markers of oxidative stress correlate with chemotherapy response and upregulation of redox enzymes, such as glutathione peroxidases, have been observed in the acquisition of chemotherapy resistance and genomic instability [reviewed in Ref. (106)].

Tubulins interact with mediators of the oxidative stress response, with direct interactions between βIII-tubulin and glutathione *S*-transferase μ4 observed in ovarian cancer cells (107). βIII-tubulin and the DNA damage repair enzyme excision repair cross-complementation group-1 (ERCC1) act together to influence patient response to taxane and paclitaxel combination treatment (108); however, the mechanisms underlying this co-operative effect are unknown.

Specific tubulin isotypes may also alter oxidative stress responses by acting as redox switches (109). In particular, ser/ala124, which is a cysteine in βIII-, βV- and βVI-tubulins, and cys239, which is a serine in βIII-, βV-, and βVI-tubulins, have been specifically identified as potential sensors of oxidative stress (109). Cys239 is readily oxidized and its oxidation inhibits microtubule assembly and stability (109). Therefore, alterations in tubulin isotype composition may influence microtubule stability in an oxidative environment to maintain microtubule integrity and cell survival in these adverse conditions. Moreover, oxidative stress influences tubulin post-translational modifications. Nitrotyrosine is a common byproduct of nitrosyl radical production in oxidative stress and can be incorporated into microtubules through the tyrosination/detyrosination cycle (110). While nitrotyrosine incorporation does not affect microtubule assembly, architecture, or cell viability (111), it does increase the stability of neuronal microtubules (112). Furthermore, elevated levels of nitrosylated α-tubulins correlate with disease stage in gliomas (113).

Oxidative stress is also induced by TBAs, suggesting an involvement of microtubules in oxidative stress responses, and is an important mechanism of action for platinum-based chemotherapeutic agents (114). Paclitaxel treatment induces reactive oxygen species through activation of the JNK pathway in melanoma cells (115). TBA treatment also influences NADPH oxidase activity, increases ROS levels and induces bystander effects in breast cancer cells (116). This effect may be mediated by changes in microtubule dynamics and stability, with these factors regulating Rac1 translocation and subsequently, NADPH oxidase activity (117, 118).

Studies in neurons and endothelial cells indicate that the microtubule cytoskeleton undergoes remodeling in response to oxidative stress (119). Oxidative stress induces microtubule depolymerization, and increases the pool of soluble tubulin (120, 121). 4-Hydroxy-2-nonenal (4-HNE), a secondary product of lipid peroxidation and marker of oxidative stress, also causes microtubule depolymerization, together with tubulin crosslinking (122, 123). This depolymerization may be caused by preferential reaction of 4-HNE with soluble tubulin, thereby disrupting the soluble/polymer fractionation of tubulin subunits and subsequent microtubule assembly (124). Interactions between microtubules and MAPs protect microtubules from depolymerization in response to oxidative stress (122, 125), and alters cellular trafficking in oxidative conditions (126).

Collectively, there is growing evidence supporting a role for tubulin isotypes and the microtubule network in both sensing and responding to oxidative stress in cancer through direct structural changes and protein–protein interactions. This is supported by observations in neuronal models, however, the specific roles of tubulin isotypes and their accessory proteins in oxidative stress responses remain to be clarified.

### Metabolic stress

Metabolic stress occurs in cancer as a result of uncontrolled cell proliferation in the absence of adequate nutrients [reviewed in Ref. (127)]. Microtubules and tubulins are involved in responding to metabolic stress by sensing and modulating metabolic processes to maintain cellular energy levels. The microtubule network is hypothesized to play a critical role in the regulation of cellular metabolism (128).

Early studies suggested that microtubules may act as a sensor of the energy state of the cell (129) with ATP depletion causing instability of detyrosinated microtubule plus ends (130, 131). AMPK is a major sensor for the metabolic state of the cell and affects microtubule dynamics by phosphorylation of CLIP170 (132). CLIP170 alters paclitaxel sensitivity in breast cancer cells by enhancing the binding of the drug to tubulin (67). In neuronal cells, activation of AMPK in metabolic stress prevents growth of axonal microtubules (133), further supporting a role for microtubules in early metabolic stress signaling events. The main neuronal tubulin, βII-tubulin, was also identified as a downstream target of AMPK in murine brain extracts (134).

Metabolic modulation of microtubule dynamics and tubulin post-translational modifications may allow for rapid and widespread stress responses. For example, nutrient starvation induces hyperacetylation of tubulin, which may act in concert with AMPK to induce autophagy in response to decreased ATP levels (77), thereby engaging multiple stress response pathways through microtubule-related signaling.

### Metabolic regulation

Tubulins and microtubules have been suspected to function as a key modulator of mitochondrial metabolism for some time (128). Recent studies have demonstrated that tubulin is capable of interacting with, and blocking the VDAC, thereby regulating ATP and metabolite compartmentalization and contributing to the Warburg effect (135–138). This interaction is mediated by the tubulin C-terminal tail (135), raising the possibility that post-translational modifications and different tubulin isotypes may differentially regulate VDAC dynamics to influence metabolic reprograming in cancer.

Tubulins, and in particular βIII-tubulin, associate with enzymes of the tricarboxylic acid cycle and glycolysis (107). *In vitro* studies in reduced systems showed that tubulin interacts with a variety of glycolytic enzymes including pyruvate kinase, phosphofructokinase, aldolase, hexokinase, GAPDH, and lactate dehydrogenase (139–144). Interactions with some of these enzymes may be isotype-specific, by interacting with the α-tubulin C-terminal tail (142) rather than the tubulin body (140).

Preferential interactions between glycolytic enzymes and either the soluble or polymerized tubulin pool may also influence metabolic activity and microtubule dynamics (139, 141, 144). GAPDH activity is differentially regulated by its interaction with either the soluble or polymerized tubulins (143), and this interaction influences microtubule dynamics (145). Interactions between metabolic enzymes and tubulins may therefore mediate bi-directional signaling events to sense and respond to metabolic stress. Indeed, mathematical modeling of metabolic pathways and tubulin’s modulation of enzyme activity suggest that glycolytic flux is regulated by microtubule polymer levels (146), however, the mechanisms by which the microtubule network influences metabolic homeostasis and the importance of the soluble and polymerized tubulin fractions in these functions remain to be characterized experimentally.

The association between GAPDH and microtubules may also influence cellular trafficking, with a recent study finding that ATP generated from vesicular GAPDH activity fuels the energy consumption of motor proteins during vesicular transport (147). Furthermore, GAPDH is known to mediate membrane fusion, and its association with microtubules may co-regulate membrane trafficking during glycolytic stress (148). The presence of GAPDH on microtubules allows the recruitment of Rab2 protein to regulate membrane and ER–Golgi trafficking independently of its catalytic activity (145, 149). Given the importance of ER–Golgi trafficking in protein glycosylation, the interaction of GAPDH with microtubules may function as a point of communication between metabolic and protein modification pathways under a range of stresses. For example, in neuronal cells, GAPDH binds tubulin through the neuronal MAP1B protein but is relocalized upon oxidative stress (150).

Specific interactions between tubulin isotypes and glycolytic enzymes support the pro-survival effect of altered tubulin isotypes in cancer. Pyruvate kinase interacts with tubulin via the tubulin C-terminal tail and depolymerizes stabilized microtubules (140, 151). In particular, βIII-tubulin interacts with the mitochondrial-localized pyruvate kinase M2 (107), which is associated with the Warburg effect. Feedback from metabolic products also influences the association of pyruvate kinase with microtubules, as well as microtubule stability (151), further supporting a role for the microtubule cytoskeleton in the regulation of metabolic flux. Altered metabolic activity also influences microtubule architecture (152), raising the possibility that the microtubule system may communicate with metabolic networks in a bi-directional manner.

βIII-tubulin has been specifically implicated in glucose stress responses. Treatment of ovarian cancer cells with tunicamycin or wortmannin to block protein glycosylation and PI3K signaling, respectively, upregulates βIII-tubulin and alters the post-translational modifications of non-mitochondrial tubulins in cell lines with low basal βIII-tubulin expression (107). βIII-tubulin induction and decreased βI-tubulin expression have also been observed for ovarian cancer cells under glucose starvation (40). Upregulation of βIII-tubulin in these conditions correlates with HuR binding to the βIII-tubulin 3′UTR (40). This function of HuR is independent of its role in the nuclear export of mRNA; however, whether HuR is involved in the stabilization of βIII-tubulin mRNA under hypoglycemic conditions was not investigated. Correlations between increased HuR, βIII-tubulin expression, and poor survival in ovarian cancer samples further support a role for this mechanism in influencing cancer progression and patient outcome (40).

The current evidence strongly supports a role for the microtubules in regulating metabolic activity and metabolic reprograming in response to nutrient starvation. However, the mechanistic details underpinning these observations is lacking and the importance of specific tubulin isotypes, tubulin post-translational modifications, and associated proteins in regulating metabolic stress responses requires further characterization.

### Autophagy

Macroautophagy (hereafter referred to as autophagy) can be induced in cells in response to diverse stresses, including metabolic and ER stress [reviewed in Ref. (153)]. Autophagy is a catabolic process that enables isolation and recycling of protein and organelle components by sequestering them into vacuoles for subsequent lysosomal degradation (154). It is also an important quality control process, allowing for the removal of damaged organelles and proteins, and protects cells from oxidative stress damage (155). Autophagic activity can support cells during ATP depletion, and thus is intrinsically linked with metabolic stress responses (154).

Recent evidence supports a role for autophagy in the survival and treatment sensitivity of cancer cells, and several recent reviews have been devoted to this topic (156–158). Microtubules have been known to play a critical role in autophagic flux for several decades (159), however our understanding of their importance in autophagy initiation, trafficking, and lysosomal fusion has been furthered in recent years.

Evidence for a microtubule role in autophagy regulation comes from the alteration of autophagic flux upon treatment with TBAs *in vitro* (160–163). Disruption of autophagic flux by TBAs is important in the mechanism of action of, and resistance to, TBAs in cancer (4, 164). The influence of TBAs on autophagy may be mediated by inhibition of Akt/mammalian target of rapamycin (mTOR) signaling (165), or suppression of microtubule dynamics, and additional studies are required to characterize this mechanism.

Microtubule-associated protein-1 light chain 3 (MAP1LC3, also referred to as LC3), a critical member of the autophagy network, interacts directly with tubulin in both its free and phosphatidylethanolamine-conjugated form (77, 160). LC3 also interacts with microtubules through MAP1 proteins (166–168). The promotion of autophagy by MAP1S reduces genomic instability to suppress tumor development in hepatocarcinoma, and MAP1S may also co-ordinate mitochondrial dynamics and autophagy (155, 167). Other autophagy proteins also associate with microtubules, including ULK1, Beclin-1, WIPI1, autophagy related (Atg) protein 5, and Atg12, which are thought to be principally involved in autophagosome formation (77, 169, 170). In neuronal models derived from neuroblastoma cells, autophagy inhibition is associated with decreased β-tubulin levels and suppressed neurite outgrowth (171). However, links between altered tubulin expression and autophagy have not yet been reported in non-neuronal cancer cells.

Autophagy initiation involves activation of the master regulator mTOR and the formation of the mTOR-containing complexes. mTOR activity is regulated by lysosomal localization (172), with mTOR associating specifically with peripheral lysosomes (173). Peripherally localized mTOR is sensitive to nutrient starvation, which causes it to be released from lysosomes to form the mTORC1 complex and initiate autophagy (172). Microtubules control the peripheral localization of lysosomes, and therefore ensure the sensitivity of mTOR to nutrient starvation (172). Spatial partitioning of the microtubule-interacting kinesins KIF2A and KIF1B between peripheral or perinuclear lysosomes also influences mTOR activation and the initiation of autophagy (173).

Microtubules act as scaffolds and sequester proteins to regulate autophagy. Activating molecule in BECN1-regulated autophagy 1 (AMBRA1) acts as a linker protein between microtubules and the PI3K signaling complex responsible for autophagy induction (169). Starvation induces phosphorylation of AMBRA1 by ULK1, releasing the Beclin-1-PI3K complex from microtubules to the ER to initiate autophagosome formation (169). Beclin-1–Bcl-2 complexes are also sequestered on microtubules during periods of high nutrient availability. JNK1-mediated phosphorylation of Bcl-2 in response to nutrient starvation causes dissociation of Beclin-1 from this complex to initiate autophagosome signaling and influence apoptosis (174). Microtubules are also involved in the transport of several proteins whose localization is required for autophagosome formation (175).

Tubulin post-translational modifications also regulate autophagy initiation, as tubulin hyperacetylation occurs before autophagosome formation in response to nutrient starvation (77). Acetylation modifications signal kinesin recruitment to microtubules, with subsequent JNK activation, and release of Beclin-1 from Beclin-1–Bcl-2 complexes to initiate autophagy (77). Therefore, tubulins serve as interacting partners in the regulation of autophagy initiation.

During autophagy initiation autophagosome membranes are produced from existing intracellular membranes and microtubules are well positioned to act as carriers of these membrane components from existing organelles to sites of phagophor nucleation. Recent studies have shown that LC3 enrichment and autophagosome formation occur at contact sites between Parkin-tagged mitochondria and the ER (176). Microtubules mediate translocation of both these organelles (177, 178) and may critically regulate their co-localization to initiate autophagosome formation.

The role of microtubules in autophagosome formation is differentially regulated in basal and starvation conditions. Microtubule dynamics are required for autophagosome formation in response to nutrient starvation (77, 162) but not under basal conditions (162, 179, 180).

Once formed, autophagosomes are transported along microtubules in both anterograde and retrograde directions (77), where they are fused with lysosomes. The role of microtubules in mediating the fusion of autophagosomes with lysosomes remains controversial. Microtubule dynamics do not affect the co-localization and fusion of autophagosomes and lysosomes (162), which can occur in the absence of microtubules (160). However, Kimura et al. argue that more efficient fusion is enabled by active transport along microtubule (181). These contrary observations may be explained by the influence of pharmacological or RNA interference-based modulators on lysosomal behavior in addition to their effects on microtubule cytoskeleton. However, studies using tools that more selectively target the autophagy machinery are required to clarify the importance of microtubules in autophagosome–lysosome fusion in autophagy, and the mechanisms regulating these processes.

Overall, microtubules regulate autophagy through scaffolding functions and in the intracellular trafficking of autophagy components. While precise mechanistic details remain elusive, it is likely that tubulin alterations seen in cancer would influence autophagic function and the ability of cells to cope with microenvironmental and chemotherapeutic stressors that cause nutrient starvation and cellular damage.

### Protein folding stress

Misfolded proteins may arise from protein damage, inadequate chaperone activity, and malfunction of protein processing systems. The ER is responsible for ensuring correct folding of membranous and secretory proteins and this organelle is highly sensitive to cellular conditions. Slight changes in any number of parameters can lead to accumulation of unfolded proteins in the ER lumen and initiation of the unfolded protein response (UPR) [reviewed in Ref. (182)]. The UPR involves the induction of the ER-associated degradation machinery that allows transport of unfolded proteins to cytoplasmic proteasomal systems, suppression of translation, and upregulation of chaperones in a concerted effort to reduce the burden of misfolded proteins (182). Initiation of the UPR leads to amelioration of ER stress, or the initiation of cell death (182). The UPR is upregulated in many cancers and is an important contributor to tumor development and maintenance (182–184). ER stress sensitizes cells to a broad range of chemotherapeutics including topoisomerase inhibitors (185), temozolomide (186), platinum-based agents (187, 188), and TBAs (189).

Glucose regulated protein 78 (GRP78) is a member of the heat shock protein 70 (Hsp70) family and a master regulator of the ER stress response (190). Alterations in GRP78 expression and localization have been linked with tumor aggressiveness, migration, and invasion as well as chemoresistance, where it acts as a pro-survival factor (182). Taxanes and vinca alkaloids induce ER stress through upregulation of GRP78 in breast cancer cells (5). ER stress is also associated with JNK activation and apoptosis, which are inhibited upon GRP78 knockdown (5, 191). GRP78 interacts with βIII-tubulin (107), however, the functional consequences of this association are unknown. These observations suggest an intrinsic link between the microtubule cytoskeleton and the initiation of ER stress responses.

Tubulin-binding agent treatment also initiates mechanisms to repress translation and ameliorate misfolded protein accumulation. Treatment of cervical cancer cells with TBAs induces P-body formation, which are cytoplasmic regions where mRNA translation is inhibited (104). P-body targeting of miRNA and mRNA is also an important regulator of numerous stress responses, including the regulation of HIF1α levels in normoxic and hypoxic conditions (192). Microtubule dynamics are also critically involved in the association of mRNA with stress granules (193), which also regulate mRNA processing in response to stress (194).

Expansion of the ER network occurs during the UPR (195), where it acts to relieve ER stress (196). Microtubules are critically involved in regulating ER morphology, trafficking, and expansion of the organelle to the periphery of the cell by direct attachment of the ER to microtubules (197). Microtubule dynamics are tightly co-regulated with ER dynamics, which are suppressed by microtubule depolymerizing agents (178, 198). ER movement can occur by attachment to the microtubule plus ends (198), or kinesin-mediated ER sliding along microtubules (58, 199). While the former mechanism occurs on highly dynamic microtubules, ER sliding occurs on acetylated microtubules (58). Therefore, tubulin post-translational modifications may act as important regulators of ER expansion during the UPR. Mitochondria are also localized to acetylated microtubules, with this PTM potentially facilitating functional ER–mitochondrial interactions with diverse consequences for the cell, including autophagy induction (58, 176). Therefore, the microtubule network may co-ordinate whole cell reprograming in response to localized ER stress.

In neuronal neuroblastoma models, collapse of the microtubule network and evolution of ubiquitinated protein aggregates at the centrosome were observed in parallel with the initiation of ER stress (200). While this suggests that maintenance of a functional ER network relies heavily upon the microtubule cytoskeleton, similar observations are yet to be reported in non-neuronal cancer cells.

These observations suggest an intrinsic link between ER homeostasis, the initiation of ER stress responses and the microtubule network; however, the mechanisms co-regulating these systems remain elusive. Improved understanding of the role of microtubules in ER function, and the importance of this organelle in tumor development and cell survival may reveal strategies for more effective use of existing treatments in cancer.

### Tubulin and molecular chaperones outside of the ER

Other chaperones outside of the ER system also interact with microtubules (201). The small heat shock protein (Hsp) α B-crystallin regulates microtubule dynamics (202) and tubulin polymerization (203) by associating with microtubules through interactions with MAPs (204). The association between α B-crystallin and tubulin may also prevent the aggregation of misfolded tubulin (202).

Heat shock protein 27 (Hsp27) associates with microtubules (205) and alters the microtubule structure by promoting microtubule nucleation distant to the centrosome (206). TBAs induce Hsp27 phosphorylation through the p38 signaling pathway in MCF-7 cells, with microtubule stabilizers and destabilizers inducing different phosphorylation patterns on this protein (207). However, the functional consequences of these phosphorylation sites are unclear. Hsp70 also associates with tubulin by interacting with the tubulin C-terminal tail, and this interaction may be mediated by MAP1B (208, 209). In particular, βIII-tubulin has been found to associate with mitochondria-localized Hsp70 (107). Hsp70 expression is induced by vinblastine treatment in melanoma cells (210). Furthermore, crosstalk between Hsp70 and oxidative stress enzymes (211) suggests that interactions between the microtubule network and these proteins could have profound implications for a variety of stress responses.

The Hsp90 family is the main cytosolic chaperones in basal and stressed conditions, where they mediate maturation of folded proteins (212). Hsp90 client proteins are diverse and include oncoproteins that promote survival in response to environmental stress [reviewed in Ref. (213)]. Hsp90 proteins have been found to associate with tubulin; however, this occurs in an ATP-independent manner, suggesting that tubulin–Hsp90 associations are not related to global tubulin re-folding or the targeting of tubulins to proteasome machinery (214, 215). The binding of Hsp90 to tubulins may instead ensure correct folding of nascent tubulin peptides, and prevent the formation of tubulin aggregates during cellular stress (214). The association between these proteins may also reflect the role of Hsp90 as a molecular chaperone for proteins translocating on microtubules (216).

Heat shock protein 90 recruitment to microtubules depends on acetylated tubulins, with HeLa cells having higher levels of acetylated tubulin and Hsp90 recruitment to microtubules compared with non-tumoral RPE1 cells (52). Tubulin acetylation is also associated with recruitment of the Hsp90 client proteins Akt and p53 to microtubules, with significant implications for downstream signaling events and chemosensitivity (52). Whether tubulin hyperacetylation is a widespread feature of cancers, or is specific to these cell types, is unclear, but these observations suggest that tubulin post-translational modifications may impact upon protein folding stress in cancer. Overall, interactions between tubulins and Hsp90 may act as an important link between tubulin PTMs, protein folding, and stress response signaling.

### Mitochondrial function

As integrators of cell state and mediators of apoptotic signaling, mitochondria play a critical role in determining cell fate in response to stress. There is growing evidence that tubulin, microtubules, and the microtubule network regulate mitochondrial function in cancer (217). Microtubules are involved in mitochondrial trafficking and degradation, with these processes influencing microtubule stability and tubulin degradation (218). Tubulin is an integral component of mitochondrial membranes (136, 137, 219), and these membranes are enriched with βIII-tubulin (107, 137, 217). Mitochondria-associated βIII-tubulin is distinguished from the cytoplasmic tubulin pool by distinct post-translational modifications (107). Interactions between tubulin and VDAC discussed above, also support a role for tubulins in mitochondrial function.

Tubulin-binding agents are known to affect mitochondrial stress (115). Microtubule stabilizing and destabilizing TBAs cause changes in the mitochondrial membrane potential, which is critical for the maintenance of respiration and regulation of apoptosis (135, 220). It is currently unclear whether these effects are independent of the tubulin-targeted activity of these agents. Nevertheless, higher levels of soluble tubulin are associated with a lower mitochondrial membrane potential in cancer cells but not in non-transformed primary cells (220). Therefore, modulation of mitochondrial function by tubulin and microtubules may influence cell stress responses and cell survival signaling in cancer.

### Cell death signaling

Failure of cellular stress responses to alleviate cellular dysfunction can result in the induction of cell death. Emerging evidence supports a role for tubulins and microtubules in the execution of cell death in response to stress. For instance, tubulins interact with regulators of mitochondrial membrane permeability and apoptosis. Interactions between tubulin, VDAC, and p53 (discussed above) may influence the mitochondrial permeability transition and regulate apoptosis induction (221). This is supported by evidence that TBAs mediate their apoptotic effects by directly compromising the mitochondrial outer membrane integrity (222), whether through interactions with their traditional target, tubulin, or with B-cell Lymphoma/Leukemia-2 (Bcl-2) (223).

Crosstalk between microtubules and apoptotic networks is also suggested by Bcl-2 involvement in TBA-mediated cell death. In leukemic cell lines, the overexpression of Bcl-2 suppresses the apoptotic response of TBAs independently of G2/M arrest and structural microtubule alterations (224–226). High Bcl-xL levels are protective against taxol-induced cell stress (225). These effects may be explained by direct interactions between Bcl-2 and tubulin (217, 227). Bcl-2 interacting mediator of cell death (Bim) is also sequestered on microtubules by binding to the dynein light chain, thereby preventing initiation of apoptotic signaling (227, 228). Once released from microtubules, Bim translocates to mitochondria, and interacts with Bcl-2, Bcl-xL, or Bax to promote apoptosis (228). Biophysical studies have also indicated that BH3-domain proteins, of which Bim is a member, can interact with tubulin through this domain (227). The pro-survival factors semaphorin 6A and survivin also associate with microtubules (107, 229, 230) with the latter affecting microtubule dynamics (229). Semaphorin 6A interacts directly with βIII-tubulin in ovarian cancer cell lines and its expression correlates with resistance to a broad range of chemotherapy agents (230). By interacting with apoptotic proteins, tubulin alterations may have a pro-survival effect by reducing the apoptotic potential of cancer cells.

Manipulation of the soluble and polymerized tubulin fractions may also modulate apoptotic potential. Bak associates with the polymerized fraction while Bid preferentially associates with the soluble fraction (227). This interaction is mediated by the β-tubulin C-terminal tail region (227), suggesting that tubulins may modulate apoptotic potential in an isotype-specific manner. However, this interaction, its tubulin isotype specificity and functional consequences are yet to be validated in the more complex cell environment.

Tubulin-binding agents are known to induce Bcl-2 phosphorylation, a state that inhibits the anti-apoptotic activity of this protein (231), suggesting that Bcl-2 activity may be regulated by microtubule integrity. However, Bcl-2 phosphorylation is elevated in cells undergoing G2/M arrest and this observation may reflect the action of TBAs on the cell cycle checkpoint, rather than apoptotic signaling (232).

Direct and indirect interactions between tubulins, apoptotic proteins, and mitochondria suggest that the microtubule network communicates with the apoptotic machinery to regulate the execution of the final stages of cell death signaling. While the precise mechanistic details of this cross-talk remain elusive, the current evidence supports a role for isotype-specific regulation of cell death by tubulins.

## Conclusion

Tubulins, microtubules, and their interacting partners are increasingly recognized as central players in the maintenance of cell homeostasis and execution of cell stress responses. Emerging evidence suggests that the modulation of tubulin isotype composition, post-translational modifications and the expression of MAPs seen in cancer influence diverse cellular functions to promote cell survival under metabolic, protein, oxidative, and hypoxic stress. Microtubules and tubulins influence protein signaling networks through molecule and organelle transport, act as scaffolds for protein–protein interactions, modulate enzyme activity, and sequester stress response mediators. Developing a detailed spatiotemporal knowledge of the specific function of tubulin isotypes, their post translation modifications and the proteins they associate with presents a major challenge, and is a necessary foundation for understanding the role of the microtubule network in the regulation and execution of stress responses.

**Figure 2:**
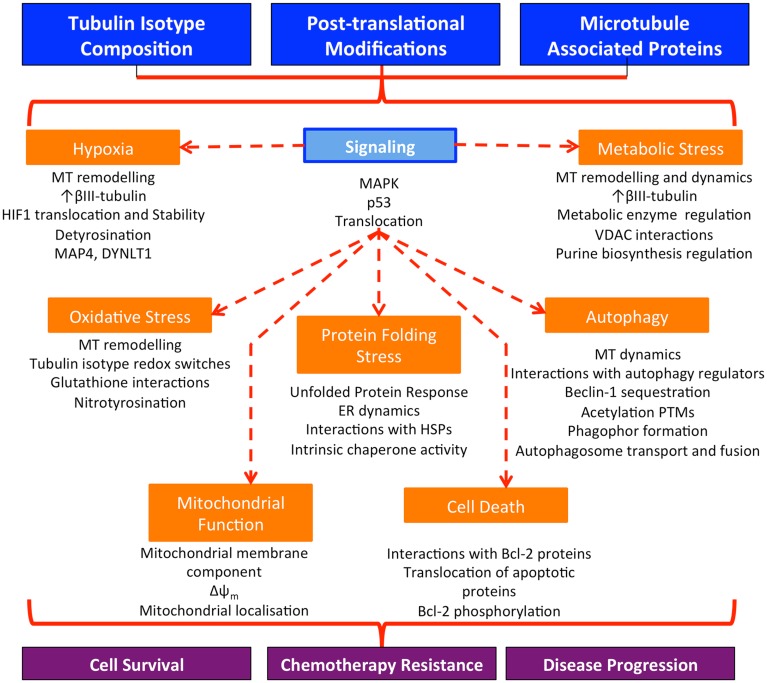
**Microtubules regulate and co-ordinate diverse cellular stress responses in cancer cells**. Alterations in the expression of tubulin isotypes, tubulin post-translational modifications, and the interaction of microtubules with MAPs seen in cancer affect a wide range of homeostatic mechanisms in response to cellular stress. Microtubules may function to co-ordinate stress responses across the cell, resulting in enhanced cell survival in the harsh tumor microenvironment, resistance to chemotherapy treatment, and the development of more aggressive disease; MT, microtubules.

By influencing a variety of cell stress responses, microtubules are well positioned to act as coordinators of cell function in response to stress. Furthermore, crosstalk between different stress response signaling events means that microtubule involvement in this context may have profound implications on diverse cellular functions (Figure 2).

Improved understanding of the role of tubulins and microtubules in cell stress responses in cancer has appreciable clinical benefits. The identification of signaling pathways influenced by the microtubule cytoskeleton may offer a source of novel anticancer treatments. A firmer grasp on the role of the microtubule cytoskeleton in cell stress responses, and in particular in chemotherapeutic stress, should also enable more effective use of existing treatments. By profiling tubulin and microtubule aberrations in tumors, chemotherapeutic combinations known to induce particular stress states could be selected to exploit altered stress response signaling in cancers. Through these avenues, a thorough understanding of the role of the microtubule cytoskeleton in stress responses has the potential to lead to larger therapeutic windows, reduced chemotherapy resistance, and more effective cancer treatment with reduced side effects.

## Conflict of Interest Statement

The authors declare that the research was conducted in the absence of any commercial or financial relationships that could be construed as a potential conflict of interest.

## References

[1] Nogales E Structural insights into microtubule function. Annu Rev Biochem (2000) 69:277–302 10.1146/annurev.biochem.69.1.277 10966460

[2] Downing KH Nogales E Tubulin structure: insights into microtubule properties and functions. Curr Opin Struct Biol (1998) 8(6):785–91 10.1016/S0959-440X(98)80099-7 9914260

[3] Jordan MA Wilson L Microtubules as a target for anticancer drugs. Nat Rev Cancer (2004) 4(4):253–65 10.1038/nrc1317 15057285

[4] Veldhoen RA Banman SL Hemmerling DR Odsen R Simmen T Simmonds AJ The chemotherapeutic agent paclitaxel inhibits autophagy through two distinct mechanisms that regulate apoptosis. Oncogene (2013) 32(6):736–46 10.1038/onc.2012.92 22430212

[5] Wang J Yin YC Hua H Li MJ Luo T Xu L Blockade of GRP78 sensitizes breast cancer cells to microtubules-interfering agents that induce the unfolded protein response. J Cell Mol Med (2009) 13(9B):3888–97 10.1111/j.1582-4934.2009.00873.x 19674193 PMC4516536

[6] Xi GM Hu XY Wu BL Jiang HM Young CY Pang YX Autophagy inhibition promotes paclitaxel-induced apoptosis in cancer cells. Cancer Lett (2011) 307(2):141–8 10.1016/j.canlet.2011.03.026 21511395

[7] Luduena RF Are tubulin isotypes functionally significant. Mol Biol Cell (1993) 4(5):445–57 10.1091/mbc.4.5.445 8334301 PMC300949

[8] Sullivan KF Cleveland DW Identification of conserved isotype-defining variable region sequences for 4 vertebrate beta-tubulin polypeptide classes. Proc Natl Acad Sci U S A (1986) 83(12):4327–31 10.1073/pnas.83.12.4327 3459176 PMC323725

[9] Janke C Bulinski JC Post-translational regulation of the microtubule cytoskeleton: mechanisms and functions. Nat Rev Mol Cell Biol (2011) 12(12):773–86 10.1038/nrm3227 22086369

[10] Kavallaris M Tait AS Walsh BJ He LF Horwitz SB Norris MD Multiple microtubule alterations are associated with vinca alkaloid resistance in human leukemia cells. Cancer Res (2001) 61(15):5803–9 11479219

[11] Martello LA Verdier-Pinard P Shen HJ He LF Torres K Orr GA Elevated levels of microtubule destabilizing factors in a taxol-resistant/dependent A549 cell line with an alpha-tubulin mutation. Cancer Res (2003) 63(6):1207–13 12649178

[12] Verdier-Pinard P Wang F Martello L Burd B Orr GA Horwitz SB Analysis of tubulin isotypes and mutations from taxol-resistant cells by combined isoelectrofocusing and mass spectrometry. Biochemistry (2003) 42(18):5349–57 10.1021/bi027293o 12731876

[13] Mozzetti S Ferlini C Concolino P Filippetti F Raspaglio G Prislei S Class III beta-tubulin overexpression is a prominent mechanism of paclitaxel resistance in ovarian cancer patients. Clin Cancer Res (2005) 11(1):298–305 15671559

[14] Sale S Sung R Shen PD Yu K Wang Y Duran GE Conservation of the class I beta-tubulin gene in human populations and lack of mutations in lung cancers and paclitaxel-resistant ovarian cancers. Mol Cancer Ther (2002) 1(3):215–25 12467216

[15] Kavallaris M Microtubules and resistance to tubulin-binding agents. Nat Rev Cancer (2010) 10(3):194–204 10.1038/nrc2803 20147901

[16] Ferrandina G Zannoni GF Martinelli E Paglia A Gallotta V Mozzetti S Class III beta-tubulin overexpression is a marker of poor clinical outcome in advanced ovarian cancer patients. Clin Cancer Res (2006) 12(9):2774–9 10.1158/1078-0432.CCR-05-2715 16675570

[17] Lee KM Cao D Itami A Pour PM Hruban RH Maitra A Class III beta-tubulin, a marker of resistance to paclitaxel, is overexpressed in pancreatic ductal adenocarcinoma and intraepithelial neoplasia. Histopathology (2007) 51(4):539–46 10.1111/j.1365-2559.2007.02792.x 17714470

[18] McCarroll JA Gan PP Liu M Kavallaris M Beta III-tubulin is a multifunctional protein involved in drug sensitivity and tumorigenesis in non-small cell lung cancer. Cancer Res (2010) 70(12):4995–5003 10.1158/0008-5472.CAN-09-4487 20501838

[19] Gan PP McCarroll JA Byrne FL Garner J Kavallaris M Specific β-tubulin isotypes can functionally enhance or diminish epothilone B sensitivity in non-small cell lung cancer cells. PLoS One (2011) 6(6):e21717 10.1371/journal.pone.0021717 21738778 PMC3126859

[20] Gan PP Kavallaris M Tubulin-targeted drug action: functional significance of class II and class IVb beta-tubulin in vinca alkaloid sensitivity. Cancer Res (2008) 68(23):9817–24 10.1158/0008-5472.CAN-08-1501 19047161

[21] Cucchiarelli V Hiser L Smith H Frankfurter A Spano A Correia JJ Beta-tubulin isotype classes II and V expression patterns in nonsmall cell lung carcinomas. Cell Motil Cytoskeleton (2008) 65(8):675–85 10.1002/cm.20297 18613117

[22] Don S Verrills NM Liaw TY Liu ML Norris MD Haber M Neuronal-associated microtubule proteins class III beta-tubulin and MAP2c in neuroblastoma: role in resistance to microtubule-targeted drugs. Mol Cancer Ther (2004) 3(9):1137–46 15367708

[23] Bhattacharya R Cabral F A ubiquitous beta-tubulin disrupts microtubule assembly and inhibits cell proliferation. Mol Biol Cell (2004) 15(7):3123–31 10.1091/mbc.E04-01-0060 15121885 PMC452570

[24] Lobert S Jefferson B Morris K Regulation of beta-tubulin isotypes by micro-RNA 100 in MCF7 breast cancer cells. Cytoskeleton (Hoboken) (2011) 68(6):355–62 10.1002/cm.20517 21634028

[25] Verrills NM Walsh BJ Cobon GS Hains PG Kavallaris M Proteome analysis of vinca alkaloid response and resistance in acute lymphoblastic leukemia reveals novel cytoskeletal alterations. J Biol Chem (2003) 278(46):45082–93 10.1074/jbc.M303378200 12949081

[26] Mozzetti S Iantomasi R De Maria I Prislei S Mariani M Camperchioli A Molecular mechanisms of patupilone resistance. Cancer Res (2008) 68(24):10197–204 10.1158/0008-5472.CAN-08-2091 19074887

[27] Dozier JH Hiser L Davis JA Thomas NS Tucci MA Benghuzzi HA Beta class II tubulin predominates in normal and tumor breast tissues. Breast Cancer Res (2003) 5(5):R157–69 10.1186/bcr631 12927047 PMC314434

[28] Blade K Menick DR Cabral F Overexpression of class I, II or IVb beta-tubulin isotypes in CHO cells is insufficient to confer resistance to paclitaxel. J Cell Sci (1999) 112(13):2213–21 10362551 10.1242/jcs.112.13.2213

[29] Ranganathan S McCauley RA Dexter DW Hudes GR Modulation of endogenous beta-tubulin isotype expression as a result of human beta(III) cDNA transfection into prostate carcinoma cells. Br J Cancer (2001) 85(5):735–40 10.1054/bjoc.2001.1956 11531260 PMC2364133

[30] Hari M Yang H Zeng C Canizales M Cabral F Expression of class III β-tubulin reduces microtubule assembly and confers resistance to paclitaxel. Cell Motil Cytoskeleton (2003) 56(1):45–56 10.1002/cm.10132 12905530

[31] Levallet G Bergot E Antoine M Creveuil C Santos AO Beau-Faller M High TUBB3 expression, an independent prognostic marker in patients with early non-small cell lung cancer treated by preoperative chemotherapy, is regulated by K-Ras signaling pathway. Mol Cancer Ther (2012) 11(5):1203–13 10.1158/1535-7163.MCT-11-0899 22411898

[32] Cochrane DR Spoelstra NS Howe EN Nordeen SK Richer JK MicroRNA-200c mitigates invasiveness and restores sensitivity to microtubule-targeting chemotherapeutic agents. Mol Cancer Ther (2009) 8(5):1055–66 10.1158/1535-7163.MCT-08-1046 19435871 PMC4573391

[33] Leskela S Leandro-Garcia LJ Mendiola M Barriuso J Inglada-Perez L Munoz I The miR-200 family controls beta-tubulin III expression and is associated with paclitaxel-based treatment response and progression-free survival in ovarian cancer patients. Endocr Relat Cancer (2011) 18(1):85–95 10.1677/ERC-10-0148 21051560

[34] Akasaka K Maesawa C Shibazaki M Maeda F Takahashi K Akasaka T Loss of class III beta-tubulin induced by histone deacetylation is associated with chemosensitivity to paclitaxel in malignant melanoma cells. J Invest Dermatol (2009) 129(6):1516–26 10.1038/jid.2008.406 19122647

[35] Izutsu N Maesawa C Shibazaki M Oikawa H Shoji T Sugiyama T Epigenetic modification is involved in aberrant expression of class III beta-tubulin, TUBB3, in ovarian cancer cells. Int J Oncol (2008) 32(6):1227–35 18497984 10.3892/ijo_32_6_1227

[36] Tsourlakis MC Weigand P Grupp K Kluth M Steurer S Schlomm T βIII-tubulin overexpression is an independent predictor of prostate cancer progression tightly linked to ERG fusion status and PTEN deletion. Am J Pathol (2014) 184(3):609–17 10.1016/j.ajpath.2013.11.007 24378408

[37] Ranganathan S Dexter DW Benetatos CA Hudes GR Cloning and sequencing of human betaIII-tubulin cDNA: induction of betaIII isotype in human prostate carcinoma cells by acute exposure to antimicrotubule agents. Biochim Biophys Acta (1998) 1395(2):237–45 10.1016/S0167-4781(97)00168-1 9473684

[38] Saussede-Aim J Matera EL Herveau S Rouault JP Ferlini C Dumontet C Vinorelbine induces beta 3-tubulin gene expression through an AP-1 site. Anticancer Res (2009) 29(8):3003–9 19661308

[39] Raspaglio G Filippetti F Prislei S Penci R De Maria I Cicchillitti L Hypoxia induces class III beta-tubulin gene expression by HIF-1 alpha binding to its 3’ flanking region. Gene (2008) 409(1–2):100–8 10.1016/j.gene.2007.11.015 18178340

[40] Raspaglio G De Maria I Filippetti F Martinelli E Zannoni GF Prislei S HuR regulates beta-tubulin isotype expression in ovarian cancer. Cancer Res (2010) 70(14):5891–900 10.1158/0008-5472.CAN-09-4656 20587520

[41] Masuda K Abdelmohsen K Gorospe M RNA-binding proteins implicated in the hypoxic response. J Cell Mol Med (2009) 13(9A):2759–69 10.1111/j.1582-4934.2009.00842.x 19583805 PMC2832090

[42] Schwarz PM Liggins JR Luduena RF Beta-tubulin isotypes purified from bovine brain have different relative stabilities. Biochemistry (1998) 37(13):4687–92 10.1021/bi972763d 9521790

[43] Boekelheide K Arcila ME Eveleth J Cis-diamminedichloroplatinum (ii) (cisplatin) alters microtubule assembly dynamics. Toxicol Appl Pharmacol (1992) 116(1):146–51 10.1016/0041-008X(92)90156-M 1529448

[44] Gan PP McCarroll JA Po’uha ST Kamath K Jordan MA Kavallaris M Microtubule dynamics, mitotic arrest, and apoptosis: drug-induced differential effects of beta III-tubulin. Mol Cancer Ther (2010) 9(5):1339–48 10.1158/1535-7163.MCT-09-0679 20442307

[45] Kumar N Flavin M Preferential action of a brain detyrosinolating carboxypeptidase on polymerized tubulin. J Biol Chem (1981) 256(14):7678–86 6114100

[46] Peris L Wagenbach M Lafanechere L Brocard J Moore AT Kozielski F Motor-dependent microtubule disassembly driven by tubulin tyrosination. J Cell Biol (2009) 185(7):1159–66 10.1083/jcb.200902142 19564401 PMC2712961

[47] Soucek K Kamaid A Phung AD Kubala L Bulinski JC Harper RW Normal and prostate cancer cells display distinct molecular profiles of α-tubulin posttranslational modifications. Prostate (2006) 66(9):954–65 10.1002/pros.20416 16541425

[48] Mialhe A Lafanechere L Treilleux I Peloux N Dumontet C Bremond A Tubulin detyrosination is a frequent occurrence in breast cancers of poor prognosis. Cancer Res (2001) 61(13):5024–7 11431336

[49] Lafanechere L Courtay-Cahen C Kawakami T Jacrot M Rudiger M Wehland J Suppression of tubulin tyrosine ligase during tumor growth. J Cell Sci (1998) 111:171–81 9405300 10.1242/jcs.111.2.171

[50] Kato C Miyazaki K Nakagawa A Ohira M Nakamura Y Ozaki T Low expression of human tubulin tyrosine ligase and suppressed tubulin tyrosination/detyrosination cycle are associated with impaired neuronal differentiation in neuroblastomas with poor prognosis. Int J Cancer (2004) 112(3):365–75 10.1002/ijc.20431 15382060

[51] Whipple RA Matrone MA Cho EH Balzer EM Vitolo MI Yoon JR Epithelial-to-mesenchymal transition promotes tubulin detyrosination and microtentacles that enhance endothelial engagement. Cancer Res (2010) 70(20):8127–37 10.1158/0008-5472.CAN-09-4613 20924103 PMC3123454

[52] Giustiniani J Daire V Cantaloube I Durand G Poüs C Perdiz D Tubulin acetylation favors Hsp90 recruitment to microtubules and stimulates the signaling function of the Hsp90 clients Akt/PKB and p53. Cell Signal (2009) 21(4):529–39 10.1016/j.cellsig.2008.12.004 19136058

[53] Zhang ZH Yamashita H Toyama T Sugiura H Omoto Y Ando Y HDAC6 expression is correlated with better survival in breast cancer. Clin Cancer Res (2004) 10(20):6962–8 10.1158/1078-0432.CCR-04-0455 15501975

[54] Li WB Zhang B Tang JH Cao Q Wu YJ Wu C Sirtuin 2, a mammalian homolog of yeast silent information regulator-2 longevity regulator, is an oligodendroglial protein that decelerates cell differentiation through deacetylating alpha-tubulin. J Neurosci (2007) 27(10):2606–16 10.1523/JNEUROSCI.4181-06.2007 17344398 PMC6672490

[55] Ng F Tang BL Sirtuins’ modulation of autophagy. J Cell Physiol (2013) 228(12):2262–70 10.1002/jcp.24399 23696314

[56] Tran AD Marmo TP Salam AA Che S Finkelstein E Kabarriti R HDAC6 deacetylation of tubulin modulates dynamics of cellular adhesions. J Cell Sci (2007) 120(8):1469–79 10.1242/jcs.03431 17389687

[57] Gao YS Hubbert CC Yao TP The microtubule-associated histone deacetylase 6 (HDAC6) regulates epidermal growth factor receptor (EGFR) endocytic trafficking and degradation. J Biol Chem (2010) 285(15):11219–26 10.1074/jbc.M109.042754 20133936 PMC2856999

[58] Friedman JR Webster BM Mastronarde DN Verhey KJ Voeltz GK ER sliding dynamics and ER-mitochondrial contacts occur on acetylated microtubules. J Cell Biol (2010) 190(3):363–75 10.1083/jcb.200911024 20696706 PMC2922647

[59] Daire V Giustiniani J Leroy-Gori I Quesnoit M Drevensek S Dimitrov A Kinesin-1 regulates microtubule dynamics via a c-Jun N-terminal kinase-dependent mechanism. J Biol Chem (2009) 284(46):31992–2001 10.1074/jbc.M109.007906 19759393 PMC2797271

[60] Miller LM Menthena A Chatterjee C Verdier-Pinard P Novikoff PM Horwitz SB Increased levels of a unique post-translationally modified βIVb-tubulin isotype in liver cancer. Biochemistry (2008) 47(28):7572–82 10.1021/bi8005225 18570381 PMC2574767

[61] Wasylyk C Zambrano A Zhao C Brants J Abecassis J Schalken JA Tubulin tyrosine ligase like 12 links to prostate cancer through tubulin posttranslational modification and chromosome ploidy. Int J Cancer (2010) 127(11):2542–53 10.1002/ijc.25261 20162578

[62] Bhat KM Setaluri V Microtubule-associated proteins as targets in cancer chemotherapy. Clin Cancer Res (2007) 13(10):2849–54 10.1158/1078-0432.CCR-06-3040 17504982

[63] Rouzier R Rajan R Wagner P Hess KR Gold DL Stec J Microtubule-associated protein tau: a marker of paclitaxel sensitivity in breast cancer. Proc Natl Acad Sci U S A (2005) 102(23):8315–20 10.1073/pnas.0408974102 15914550 PMC1149405

[64] Soltani MH Pichardo R Song ZQ Sangha N Camacho F Satyamoorthy K Microtubule-associated protein 2, a marker of neuronal differentiation, induces mitotic defects, inhibits growth of melanoma cells, and predicts metastatic potential of cutaneous melanoma. Am J Pathol (2005) 166(6):1841–50 10.1016/S0002-9440(10)62493-5 15920168 PMC1602405

[65] Song ZQ He CD Sun CK Xu YN Jin X Zhang Y Increased expression of MAP2 inhibits melanoma cell proliferation, invasion and tumor growth in vitro and in vivo. Exp Dermatol (2010) 19(11):958–64 10.1111/j.1600-0625.2009.01020.x 20100193

[66] Verrills NM Flemming CL Liu M Ivery MT Cobon GS Norris MD Microtubule alterations and mutations induced by desoxyepothilone B: implications for drug-target interactions. Chem Biol (2003) 10(7):597–607 10.1016/S1074-5521(03)00141-8 12890533

[67] Sun X Li D Yang Y Ren Y Li J Wang Z Microtubule-binding protein CLIP-170 is a mediator of paclitaxel sensitivity. J Pathol (2012) 226(4):666–73 10.1002/path.3026 21989536

[68] Byrne FL Yang L Phillips PA Hansford LM Fletcher JI Ormandy CJ RNAi-mediated stathmin suppression reduces lung metastasis in an orthotopic neuroblastoma mouse model. Oncogene (2014) 33(7):882–90 10.1038/onc.2013.11 23396365

[69] Rath O Kozielski F Kinesins and cancer. Nat Rev Cancer (2012) 12(8):527–39 10.1038/nrc3310 22825217

[70] Sirajuddin M Rice LM Vale RD Regulation of microtubule motors by tubulin isotypes and post-translational modifications. Nat Cell Biol (2014) 16(4):335–44 10.1038/ncb2920 24633327 PMC4117587

[71] LaPointe NE Morfini G Brady ST Feinstein SC Wilson L Jordan MA Effects of eribulin, vincristine, paclitaxel and ixabepilone on fast axonal transport and kinesin-1 driven microtubule gliding: implications for chemotherapy-induced peripheral neuropathy. Neurotoxicology (2013) 37(0):231–9 10.1016/j.neuro.2013.05.008 23711742 PMC4169189

[72] Dhillon AS Hagan S Rath O Kolch W MAP kinase signalling pathways in cancer. Oncogene (2007) 26(22):3279–90 10.1038/sj.onc.1210421 17496922

[73] Reszka AA Seger R Diltz CD Krebs EG Fischer EH Association of mitogen-activated protein-kinase with the microtubule cytoskeleton. Proc Natl Acad Sci U S A (1995) 92(19):8881–5 10.1073/pnas.92.19.8881 7568036 PMC41071

[74] Zhou J Gupta K Yao J Ye KQ Panda D Giannakakou P Paclitaxel-resistant human ovarian cancer cells undergo c-Jun NH2-terminal kinase-mediated apoptosis in response to noscapine. J Biol Chem (2002) 277(42):39777–85 10.1074/jbc.M203927200 12183452

[75] Wang TH Popp DM Wang HS Saitoh M Mural JG Henley DC Microtubule dysfunction induced by paclitaxel initiates apoptosis through both c-Jun N-terminal kinase (JNK)-dependent and -independent pathways in ovarian cancer cells. J Biol Chem (1999) 274(12):8208–16 10.1074/jbc.274.12.8208 10075725

[76] Seidman R Gitelman I Sagi O Horwitz SB Wolfson M The role of ERK 1/2 and p38 MAP-kinase pathways in taxol-induced apoptosis in human ovarian carcinoma cells. Exp Cell Res (2001) 268(1):84–92 10.1006/excr.2001.5262 11461121

[77] Geeraert C Ratier A Pfisterer SG Perdiz D Cantaloube I Rouault A Starvation-induced hyperacetylation of tubulin is required for the stimulation of autophagy by nutrient deprivation. J Biol Chem (2010) 285(31):24184–94 10.1074/jbc.M109.091553 20484055 PMC2911293

[78] Tararuk T Ostman N Li WR Bjorkblom B Padzik A Zdrojewska J JNK1 phosphorylation of SCG10 determines microtubule dynamics and axodendritic length. J Cell Biol (2006) 173(2):265–77 10.1083/jcb.200511055 16618812 PMC2063817

[79] Chang LF Jones Y Ellisman MH Goldstein LS Karin M JNK1 is required for maintenance of neuronal microtubules and controls phosphorylation of microtubule-associated proteins. Dev Cell (2003) 4(4):521–33 10.1016/S1534-5807(03)00094-7 12689591

[80] Fan M Chambers TC Role of mitogen-activated protein kinases in the response of tumor cells to chemotherapy. Drug Resist Updat (2001) 4(4):253–67 10.1054/drup.2001.0214 11991680

[81] Brantley-Finley C Lyle CS Du LH Goodwin ME Hall T Szwedo D The JNK, ERK and p53 pathways play distinct roles in apoptosis mediated by the antitumor agents vinblastine, doxorubicin, and etoposide. Biochem Pharmacol (2003) 66(3):459–69 10.1016/S0006-2952(03)00255-7 12907245

[82] Kolomeichuk SN Terrano DT Lyle CS Sabapathy K Chambers TC Distinct signaling pathways of microtubule inhibitors – vinblastine and taxol induce JNK-dependent cell death but through AP-1-dependent and AP-1-independent mechanisms, respectively. FEBS J (2008) 275(8):1889–99 10.1111/j.1742-4658.2008.06349.x 18341588

[83] Hoshi M Ohta K Gotoh Y Mori A Murofushi H Sakai H Mitogen-activated-protein-kinase-catalyzed phosphorylation of microtubule-associated proteins, microtubule-associated protein-2 and microtubule-associated protein-4, induces an alteration in their function. Eur J Biochem (1992) 203(1–2):43–52 10.1111/j.1432-1033.1992.tb19825.x 1309698

[84] Reszka AA Bulinski JC Krebs EG Fischer EH Mitogen-activated protein kinase extracellular signal-regulated kinase 2 regulates cytoskeletal organization and chemotaxis via catalytic and microtubule-specific interactions. Mol Biol Cell (1997) 8(7):1219–32 10.1091/mbc.8.7.1219 9243503 PMC276148

[85] Hu JY Chu ZG Han J Dang YM Yan H Zhang Q The p38/MAPK pathway regulates microtubule polymerization through phosphorylation of MAP4 and Op18 in hypoxic cells. Cell Mol Life Sci (2010) 67(2):321–33 10.1007/s00018-009-0187-z 19915797 PMC11115776

[86] Liu XM Wang LG Kreis W Budman DR Adams LM Differential effect of vinorelbine versus paclitaxel on ERK2 kinase activity during apoptosis in MCF-7 cells. Br J Cancer (2001) 85(9):1403–11 10.1054/bjoc.2001.1910 11720482 PMC2375254

[87] Giannakakou P Nakano M Nicolaou KC O’Brate A Yu I Blagosklonny MV Enhanced microtubule-dependent trafficking and p53 nuclear accumulation by suppression of microtubule dynamics. Proc Natl Acad Sci U S A (2002) 99(16):10855–60 10.1073/pnas.132275599 12145320 PMC125062

[88] Galigniana MD Harrell JM O’Hagen HM Ljungman M Pratt WB Hsp90-binding immunophilins link p53 to dynein during p53 transport to the nucleus. J Biol Chem (2004) 279(21):22483–9 10.1074/jbc.M402223200 15004035

[89] Giannakakou P Sackett DL Ward Y Webster KR Blagosklonny MV Fojo T p53 Is associated with cellular microtubules and is transported to the nucleus by dynein. Nat Cell Biol (2000) 2(10):709–17 10.1038/35036335 11025661

[90] Peng YH Chen LH Li CG Lu WG Chen JD Inhibition of MDM2 by hsp90 contributes to mutant p53 stabilization. J Biol Chem (2001) 276(44):40583–90 10.1074/jbc.M102817200 11507088

[91] Kim JH Yoon EK Chung HJ Park SY Hong KM Lee CH p53 Acetylation enhances taxol-induced apoptosis in human cancer cells. Apoptosis (2013) 18(1):110–20 10.1007/s10495-012-0772-8 23161364

[92] Lee SY Kim JW Jeong MH An JH Jang SM Song KH Microtubule-associated protein 1B light chain (MAP1B-LC1) negatively regulates the activity of tumor suppressor p53 in neuroblastoma cells. FEBS Lett (2008) 582(19):2826–32 10.1016/j.febslet.2008.07.021 18656471

[93] Galmarini CM Kamath K Vanier-Viornery A Hervieu V Peiller E Falette N Drug resistance associated with loss of p53 involves extensive alterations in microtubule composition and dynamics. Br J Cancer (2003) 88(11):1793–9 10.1038/sj.bjc.6600960 12771997 PMC2377136

[94] Mabjeesh NJ Escuin D LaVallee TM Pribluda VS Swartz GM Johnson MS 2ME2 inhibits tumor growth and angiogenesis by disrupting microtubules and dysregulating HIF. Cancer Cell (2003) 3(4):363–75 10.1016/S1535-6108(03)00077-1 12726862

[95] Obacz J Pastorekova S Vojtesek B Hrstka R Cross-talk between HIF and p53 as mediators of molecular responses to physiological and genotoxic stresses. Mol Cancer (2013) 12(1):93 10.1186/1476-4598-12-93 23945296 PMC3844392

[96] Ivan M Kondo K Yang HF Kim W Valiando J Ohh M HIF alpha targeted for VHL-mediated destruction by proline hydroxylation: implications for O-2 sensing. Science (2001) 292(5516):464–8 10.1126/science.1059817 11292862

[97] Fang YD Xu X Dang YM Zhang YM Zhang JP Hu JY MAP4 mechanism that stabilizes mitochondrial permeability transition in hypoxia: microtubule enhancement and DYNLT1 interaction with VDAC1. PLoS One (2011) 6(12):e28052 10.1371/journal.pone.0028052 22164227 PMC3229508

[98] Yoon SO Shin S Mercurio AM Hypoxia stimulates carcinoma invasion by stabilizing microtubules and promoting the Rab11 trafficking of the alpha 6 beta 4 integrin. Cancer Res (2005) 65(7):2761–9 10.1158/0008-5472.CAN-04-4122 15805276

[99] Xu X Zhang Q Hu JY Zhang DX Jiang XP Jia JZ Phosphorylation of DYNLT1 at serine 82 regulates microtubule stability and mitochondrial permeabilization in hypoxia. Mol Cells (2013) 36(4):322–32 10.1007/s10059-013-0114-x 24170091 PMC3887991

[100] Hinman MN Lou H Diverse molecular functions of Hu proteins. Cell Mol Life Sci (2008) 65(20):3168–81 10.1007/s00018-008-8252-6 18581050 PMC2580827

[101] Katsetos CD Del Valle L Geddes JF Assimakopoulou M Legido A Boyd JC Aberrant localization of the neuronal class III beta-tubulin in astrocytomas – a marker for anaplastic potential. Arch Pathol Lab Med (2001) 125(5):613–24 11300931 10.5858/2001-125-0613-ALOTNC

[102] Frew IJ Smole Z Thoma CR Krek W Genetic deletion of the long isoform of the von Hippel-Lindau tumour suppressor gene product alters microtubule dynamics. Eur J Cancer (2013) 49(10):2433–40 10.1016/j.ejca.2013.02.024 23541568

[103] Carbonaro M Escuin D O’Brate A Thadani-Mulero M Giannakakou P Microtubules regulate hypoxia-inducible factor-1 alpha protein trafficking and activity implications for taxane therapy. J Biol Chem (2012) 287(15):11859–69 10.1074/jbc.M112.345587 22367210 PMC3320934

[104] Carbonaro M O’Brate A Giannakakou P Microtubule disruption targets HIF-1 alpha mRNA to cytoplasmic P-bodies for translational repression. J Cell Biol (2011) 192(1):83–99 10.1083/jcb.201004145 21220510 PMC3019555

[105] Al-Mehdi AB Pastukh VM Swiger BM Reed DJ Patel MR Bardwell GC Perinuclear mitochondrial clustering creates an oxidant-rich nuclear domain required for hypoxia-induced transcription. Sci Signal (2012) 5(231):ra47 10.1126/scisignal.2002712 22763339 PMC3565837

[106] Gorrini C Harris IS Mak TW Modulation of oxidative stress as an anticancer strategy. Nat Rev Drug Discov (2013) 12(12):931–47 10.1038/nrd4002 24287781

[107] Cicchillitti L Penci R Di Michele M Filippetti F Rotilio D Donati MB Proteomic characterization of cytoskeletal and mitochondrial class III beta-tubulin. Mol Cancer Ther (2008) 7(7):2070–9 10.1158/1535-7163.MCT-07-2370 18645017

[108] Azuma K Sasada T Kawahara A Takamori S Hattori S Ikeda J Expression of ERCC1 and class III beta-tubulin in non-small cell lung cancer patients treated with carboplatin and paclitaxel. Lung Cancer (2009) 64(3):326–33 10.1016/j.lungcan.2008.09.002 18977553

[109] Joe PA Banerjee A Luduena RF The roles of cys124 and ser239 in the functional properties of human beta III tubulin. Cell Motil Cytoskeleton (2008) 65(6):476–86 10.1002/cm.20274 18435451

[110] Eiserich JP Estevez AG Bamberg TV Ye YZ Chumley PH Beckman JS Microtubule dysfunction by posttranslational nitrotyrosination of alpha-tubulin: a nitric oxide-dependent mechanism of cellular injury. Proc Natl Acad Sci U S A (1999) 96(11):6365–70 10.1073/pnas.96.11.6365 10339593 PMC26887

[111] Bisig CG Purro SA Contin MA Barra HS Arce CA Incorporation of 3-nitrotyrosine into the C-terminus of alpha-tubulin is reversible and not detrimental to dividing cells. Eur J Biochem (2002) 269(20):5037–45 10.1046/j.1432-1033.2002.03220.x 12383263

[112] Cappelletti G Maggioni MG Ronchi C Maci R Tedeschi G Protein tyrosine nitration is associated with cold- and drug-resistant microtubules in neuronal-like PC 12 cells. Neurosci Lett (2006) 401(1–2):159–64 10.1016/j.neulet.2006.03.009 16567039

[113] Fiore G Di Cristo C Monti G Amoresano A Columbano L Pucci P Tubulin nitration in human gliomas. Neurosci Lett (2006) 394(1):57–62 10.1016/j.neulet.2005.10.011 16257120

[114] Kim JS Lee JH Jeong WW Choi DH Cha HJ Kim DH Reactive oxygen species-dependent EndoG release mediates cisplatin-induced caspase-independent apoptosis in human head and neck squamous carcinoma cells. Int J Cancer (2008) 122(3):672–80 10.1002/ijc.23158 17955488

[115] Selimovic D Hassan M Haikel Y Hengge UR Taxol-induced mitochondrial stress in melanoma cells is mediated by activation of c-Jun N-terminal kinase (JNK) and p38 pathways via uncoupling protein 2. Cell Signal (2008) 20(2):311–22 10.1016/j.cellsig.2007.10.015 18068334

[116] Alexandre J Hu Y Lu W Pelicano H Huang P Novel action of paclitaxel against cancer cells: bystander effect mediated by reactive oxygen species. Cancer Res (2007) 67(8):3512–7 10.1158/0008-5472.CAN-06-3914 17440056

[117] Putnam AJ Cunningham JJ Pillemer BB Mooney DJ External mechanical strain regulates membrane targeting of Rho GTPases by controlling microtubule assembly. Am J Physiol Cell Physiol (2003) 284(3):C627–39 10.1152/ajpcell.00137.2002 12409284

[118] Cheng G Diebold BA Hughes Y Lambeth JD Nox1-dependent reactive oxygen generation is regulated by Rac1. J Biol Chem (2006) 281(26):17718–26 10.1074/jbc.M512751200 16636067

[119] Roediger B Armati PJ Oxidative stress induces axonal beading in cultured human brain tissue. Neurobiol Dis (2003) 13(3):222–9 10.1016/S0969-9961(03)00038-X 12901836

[120] Valen G Sonden A Vaage J Malm E Kjellstrom BT Hydrogen peroxide induces endothelial cell atypia and cytoskeleton depolymerization. Free Radic Biol Med (1999) 26(11–12):1480–8 10.1016/S0891-5849(99)00009-X 10401612

[121] Hinshaw DB Miller MT Omann GM Beals TF Hyslop PA A cellular-model of oxidant-mediated neuronal injury. Brain Res (1993) 615(1):13–26 10.1016/0006-8993(93)91110-E 8364721

[122] Stewart BJ Doorn JA Petersen DR Residue-specific adduction of tubulin by 4-hydroxynonenal and 4-oxononenal causes cross-linking and inhibits polymerization. Chem Res Toxicol (2007) 20(8):1111–9 10.1021/tx700106v 17630713

[123] Neely MD Boutte A Milatovic D Montine TJ Mechanisms of 4-hydroxynonenal-induced neuronal microtubule dysfunction. Brain Res (2005) 1037(1–2):90–8 10.1016/j.brainres.2004.12.027 15777756

[124] Kokubo J Nagatani N Hiroki K Kuroiwa K Watanabe N Arai T Mechanism of destruction of microtubule structures by 4-hydroxy-2-nonenal. Cell Struct Funct (2008) 33(1):51–9 10.1247/csf.07038 18360009

[125] Divinski I Holtser-Cochav M Vulih-Schultzman I Steingart RA Gozes I Peptide neuroprotection through specific interaction with brain tubulin. J Neurochem (2006) 98(3):973–84 10.1111/j.1471-4159.2006.03936.x 16893427

[126] Stamer K Vogel R Thies E Mandelkow E Mandelkow EM Tau blocks traffic of organelles, neurofilaments, and APP vesicles in neurons and enhances oxidative stress. J Cell Biol (2002) 156(6):1051–63 10.1083/jcb.200108057 11901170 PMC2173473

[127] Altman BJ Rathmell JC Metabolic stress in autophagy and cell death pathways. Cold Spring Harb Perspect Biol (2012) 4(9):a008763 10.1101/cshperspect.a008763 22952396 PMC3428762

[128] Saks VA Kuznetsov AV Khuchua ZA Vasilyeva EV Belikova JO Kesvatera T Control of cellular respiration in-vivo by mitochondrial outer-membrane and by creatine-kinase – a new speculative hypothesis – possible involvement of mitochondrial-cytoskeleton interactions. J Mol Cell Cardiol (1995) 27(1):625–45 10.1016/S0022-2828(08)80056-9 7760382

[129] Bershadsky AD Gelfand VI ATP-dependent regulation of cytoplasmic microtubule disassembly. Proc Natl Acad Sci U S A (1981) 78(6):3610–3 10.1073/pnas.78.6.3610 6943561 PMC319620

[130] Infante AS Stein MS Zhai Y Borisy GG Gundersen GG Detyrosinated (Glu) microtubules are stabilized by an ATP-sensitive plus-end cap. J Cell Sci (2000) 113(22):3907–19 11058078 10.1242/jcs.113.22.3907

[131] Marcussen M Larsen PJ Cell cycle-dependent regulation of cellular ATP concentration, and depolymerization of the interphase microtubular network induced by elevated cellular ATP concentration in whole fibroblasts. Cell Motil Cytoskeleton (1996) 35(2):94–9 10.1002/(SICI)1097-0169(1996)35:2<94::AID-CM2>3.0.CO;2-I 8894279

[132] Nakano A Kato H Watanabe T Min KD Yamazaki S Asano Y AMPK controls the speed of microtubule polymerization and directional cell migration through CLIP-170 phosphorylation. Nat Cell Biol (2010) 12(6):583–U139 10.1038/ncb2060 20495555

[133] Williams T Courchet J Viollet B Brenman JE Polleux F AMP-activated protein kinase (AMPK) activity is not required for neuronal development but regulates axogenesis during metabolic stress. Proc Natl Acad Sci U S A (2011) 108(14):5849–54 10.1073/pnas.1013660108 21436046 PMC3078367

[134] Tuerk RD Thali RF Auchli Y Rechsteiner H Brunisholz RA Schlattner U New candidate targets of AMP-activated protein kinase in murine brain revealed by a novel multidimensional substrate-screen for protein kinases. J Proteome Res (2007) 6(8):3266–77 10.1021/pr070160a 17608512

[135] Sheldon KL Maldonado EN Lemasters JJ Rostovtseva TK Bezrukov SM Phosphorylation of voltage-dependent anion channel by serine/threonine kinases governs its interaction with tubulin. PLoS One (2011) 6(10):e25539 10.1371/journal.pone.0025539 22022409 PMC3192757

[136] Rostovtseva TK Sheldon KL Hassanzadeh E Monge C Saks V Bezrukov SM Tubulin binding blocks mitochondrial voltage-dependent anion channel and regulates respiration. Proc Natl Acad Sci U S A (2008) 105(48):18746–51 10.1073/pnas.0806303105 19033201 PMC2596221

[137] Carre M Andre N Carles G Borghi H Brichese L Briand C Tubulin is an inherent component of mitochondrial membranes that interacts with the voltage-dependent anion channel. J Biol Chem (2002) 277(37):33664–9 10.1074/jbc.M203834200 12087096

[138] Maldonado EN Sheldon KL DeHart DN Patnaik J Manevich Y Townsend DM Voltage-dependent anion channels modulate mitochondrial metabolism in cancer cells: regulation by free tubulin and erastin. J Biol Chem (2013) 288(17):11920–9 10.1074/jbc.M112.433847 23471966 PMC3636879

[139] Vertessy BG Bankfalvi D Kovacs J Low P Lehotzky A Ovadi J Pyruvate kinase as a microtubule destabilizing factor in vitro. Biochem Biophys Res Commun (1999) 254(2):430–5 10.1006/bbrc.1998.9957 9918855

[140] Orosz F Santamaria B Ovadi J Aragon JJ Phosphofructokinase from *Dictyostelium discoideum* is a potent inhibitor of tubulin polymerization. Biochemistry (1999) 38(6):1857–65 10.1021/bi981350p 10026266

[141] Marmillot P Keith T Srivastava DK Knull HR Effect of tubulin on the activity of the muscle isoenzyme of lactate-dehydrogenase. Arch Biochem Biophys (1994) 315(2):467–72 10.1006/abbi.1994.1526 7986093

[142] Volker KW Knull HR A glycolytic enzyme binding domain on tubulin. Arch Biochem Biophys (1997) 338(2):237–43 10.1006/abbi.1996.9819 9028878

[143] Muronetz VI Wang ZX Keith TJ Knull HR Srivastava DK Binding constants and stoichiometries of glyceraldehyde-3-phosphate dehydrogenase-tubulin complexes. Arch Biochem Biophys (1994) 313(2):253–60 10.1006/abbi.1994.1385 8080270

[144] Durrieu C Berniervalentin F Rousset B Microtubules bind glyceraldehyde-3-phosphate dehydrogenase and modulate its enzyme-activity and quaternary structure. Arch Biochem Biophys (1987) 252(1):32–40 10.1016/0003-9861(87)90005-1 3813539

[145] Tisdale EJ Kelly C Artalejo CR Glyceraldehyde-3-phosphate dehydrogenase interacts with Rab2 and plays an essential role in endoplasmic reticulum to Golgi transport exclusive of its glycolytic activity. J Biol Chem (2004) 279(52):54046–52 10.1074/jbc.M409472200 15485821

[146] Aon MA Cortassa S Coherent and robust modulation of a metabolic network by cytoskeletal organization and dynamics. Biophys Chem (2002) 97(2–3):213–31 10.1016/S0301-4622(02)00056-X 12050011

[147] Zala D Hinckelmann MV Yu H da Cunha MM Liot G Cordelieres FP Vesicular glycolysis provides on-board energy for fast axonal transport. Cell (2013) 152(3):479–91 10.1016/j.cell.2012.12.029 23374344

[148] Glaser PE Han XL Gross RW Tubulin is the endogenous inhibitor of the glyceraldehyde 3-phosphate dehydrogenase isoform that catalyzes membrane fusion: implications for the coordinated regulation of glycolysis and membrane fusion. Proc Natl Acad Sci U S A (2002) 99(22):14104–9 10.1073/pnas.222542999 12381782 PMC137844

[149] Tisdale EJ Azizi F Artalejo CR Rab2 utilizes glyceraldehyde-3-phosphate dehydrogenase and protein kinase c iota to associate with microtubules and to recruit dynein. J Biol Chem (2009) 284(9):5876–84 10.1074/jbc.M807756200 19106097 PMC2645835

[150] Cueille N Blanc CT Riederer IM Riederer BM Microtubule-associated protein 1B binds glyceraldehyde-3-phosphate dehydrogenase. J Proteome Res (2007) 6(7):2640–7 10.1021/pr070081z 17521179

[151] Kovacs J Low P Pacz A Horvath I Olah J Ovadi J Phosphoenolpyruvate-dependent tubulin-pyruvate kinase interaction at different organizational levels. J Biol Chem (2003) 278(9):7126–30 10.1074/jbc.M210244200 12482859

[152] Brinkley BR Barham SS Barranco SC Fuller GM Rotenone inhibition of spindle microtubule assembly in mammalian-cells. Exp Cell Res (1974) 85(1):41–6 10.1016/0014-4827(74)90210-9 4857086

[153] Dodson M Darley-Usmar V Zhang JH Cellular metabolic and autophagic pathways: traffic control by redox signaling. Free Radic Biol Med (2013) 63:207–21 10.1016/j.freeradbiomed.2013.05.014 23702245 PMC3729625

[154] Jin SK White E Role of autophagy in cancer – management of metabolic stress. Autophagy (2007) 3(1):28–31 16969128 10.4161/auto.3269 PMC2770734

[155] Xie R Wang F McKeehan WL Liu LY Autophagy enhanced by microtubule- and mitochondrion-associated MAP1S suppresses genome instability and hepatocarcinogenesis. Cancer Res (2011) 71(24):7537–46 10.1158/0008-5472.CAN-11-2170 22037873 PMC3242898

[156] Guo JY Xia B White E Autophagy-mediated tumor promotion. Cell (2013) 155(6):1216–9 10.1016/j.cell.2013.11.019 24315093 PMC3987898

[157] Sui X Chen R Wang Z Huang Z Kong N Zhang M Autophagy and chemotherapy resistance: a promising therapeutic target for cancer treatment. Cell Death Dis (2013) 4:e838 10.1038/cddis.2013.350 24113172 PMC3824660

[158] Macintosh RL Ryan KM Autophagy in tumour cell death. Semin Cancer Biol (2013) 23(5):344–51 10.1016/j.semcancer.2013.05.006 23774296

[159] Amenta JS Sargus MJ Baccino FM Effect of microtubular or translational inhibitors on general cell protein degradation – evidence for a dual catabolic pathway. Biochem J (1977) 168(2):223–7 597269 10.1042/bj1680223 PMC1183755

[160] Fass E Shvets E Degani I Hirschberg K Elazar Z Microtubules support production of starvation-induced autophagosomes but not their targeting and fusion with lysosomes. J Biol Chem (2006) 281(47):36303–16 10.1074/jbc.M607031200 16963441

[161] Fengsrud M Roos N Berg T Liou WL Slot JW Seglen PO Ultrastructural and immunocytochemical characterization of autophagic vacuoles in isolated hepatocytes – effects of vinblastine and asparagine on vacuole distributions. Exp Cell Res (1995) 221(2):504–19 10.1006/excr.1995.1402 7493651

[162] Kochl R Hu XW Chan EY Tooze SA Microtubules facilitate autophagosome formation and fusion of autophagosomes with endosomes. Traffic (2006) 7(2):129–45 10.1111/j.1600-0854.2005.00368.x 16420522

[163] Webb JL Ravikumar B Rubinsztein DC Microtubule disruption inhibits autophagosome-lysosome fusion: implications for studying the roles of aggresomes in polyglutamine diseases. Int J Biochem Cell Biol (2004) 36(12):2541–50 10.1016/j.biocel.2004.02.003 15325591

[164] Acharya BR Bhattacharyya S Choudhury D Chakrabarti G The microtubule depolymerizing agent naphthazarin induces both apoptosis and autophagy in A549 lung cancer cells. Apoptosis (2011) 16(9):924–39 10.1007/s10495-011-0613-1 21667044

[165] Viola G Bortolozzi R Hamel E Moro S Brun P Castagliuolo I MG-2477, a new tubulin inhibitor, induces autophagy through inhibition of the Akt/mTOR pathway and delayed apoptosis in A549 cells. Biochem Pharmacol (2012) 83(1):16–26 10.1016/j.bcp.2011.09.017 21964343 PMC3234688

[166] Kouno T Mizuguchi M Tanida I Ueno T Kanematsu T Mori Y Solution structure of microtubule-associated protein light chain 3 and identification of its functional subdomains. J Biol Chem (2005) 280(26):24610–7 10.1074/jbc.M413565200 15857831

[167] Liu LY McKeehan WL Wang F Xie R MAP1S enhances autophagy to suppress tumorigenesis. Autophagy (2012) 8(2):278–80 10.4161/auto.8.2.18939 22301994 PMC3336082

[168] Xie R Nguyen S McKeehan K Wang F McKeehan WL Liu LY Microtubule-associated protein 1S (MAP1S) bridges autophagic components with microtubules and mitochondria to affect autophagosomal biogenesis and degradation. J Biol Chem (2011) 286(12):10367–77 10.1074/jbc.M110.206532 21262964 PMC3060490

[169] Di Bartolomeo S Corazzari M Nazio F Oliverio S Lisi G Antonioli M The dynamic interaction of AMBRA1 with the dynein motor complex regulates mammalian autophagy. J Cell Biol (2010) 191(1):155–68 10.1083/jcb.201002100 20921139 PMC2953445

[170] Luo SQ Garcia-Arencibia M Zhao R Puri C Toh PP Sadiq O Bim inhibits autophagy by recruiting beclin 1 to microtubules. Mol Cell (2012) 47(3):359–70 10.1016/j.molcel.2012.05.040 22742832 PMC3419265

[171] Chen JX Sun YJ Wang P Long DX Li W Li L Induction of autophagy by TOCP in differentiated human neuroblastoma cells lead to degradation of cytoskeletal components and inhibition of neurite outgrowth. Toxicology (2013) 310:92–7 10.1016/j.tox.2013.05.012 23743148

[172] Sancak Y Bar-Peled L Zoncu R Markhard AL Nada S Sabatini DM Ragulator-Rag complex targets mTORC1 to the lysosomal surface and is necessary for its activation by amino acids. Cell (2010) 141(2):290–303 10.1016/j.cell.2010.02.024 20381137 PMC3024592

[173] Korolchuk VI Saiki S Lichtenberg M Siddiqi FH Roberts EA Imarisio S Lysosomal positioning coordinates cellular nutrient responses. Nat Cell Biol (2011) 13(4):453–U242 10.1038/ncb2204 21394080 PMC3071334

[174] Wei YJ Pattingre S Sinha S Bassik M Levine B JNK1-mediated phosphorylation of BcI-2 regulates starvation-induced autophagy. Mol Cell (2008) 30(6):678–88 10.1016/j.molcel.2008.06.001 18570871 PMC2478643

[175] Mochizuki Y Ohashi R Kawamura T Iwanari H Kodama T Naito M Phosphatidylinositol 3-phosphatase myotubularin-related protein 6 (MTMR6) is regulated by small GTPase Rab1B in the early secretory and autophagic pathways. J Biol Chem (2013) 288(2):1009–21 10.1074/jbc.M112.395087 23188820 PMC3542987

[176] Yang JY Yang WY Bit-by-bit autophagic removal of parkin-labelled mitochondria. Nat Commun (2013) 4:2428 10.1038/ncomms3428 24013556

[177] Appaix F Kuznetsov AV Usson Y Kay L Andrienko T Olivares J Possible role of cytoskeleton in intracellular arrangement and regulation of mitochondria. Exp Physiol (2003) 88(1):175–90 10.1113/eph8802511 12525866

[178] Terasaki M Chen LB Fujiwara K Microtubules and the endoplasmic-reticulum are highly interdependent structures. J Cell Biol (1986) 103(4):1557–68 10.1083/jcb.103.4.1557 3533956 PMC2114338

[179] Aplin A Jasionowski T Tuttle DL Lenk SE Dunn WA Cytoskeletal elements are required for the formation and maturation of autophagic vacuoles. J Cell Physiol (1992) 152(3):458–66 10.1002/jcp.1041520304 1506410

[180] Reunanen H Marttinen M Hirsimaki P Effects of griseofulvin and nocodazole on the accumulation of autophagic vacuoles in ehrlich ascites tumor-cells. Exp Mol Pathol (1988) 48(1):97–102 10.1016/0014-4800(88)90048-2 3335254

[181] Kimura S Noda T Yoshimori T Dynein-dependent movement of autophagosomes mediates efficient encounters with lysosomes. Cell Struct Funct (2008) 33(1):109–22 10.1247/csf.08005 18388399

[182] Luo B Lee AS The critical roles of endoplasmic reticulum chaperones and unfolded protein response in tumorigenesis and anticancer therapies. Oncogene (2013) 32(7):805–18 10.1038/onc.2012.130 22508478 PMC3819728

[183] Ma YJ Hendershot LM The role of the unfolded protein response in tumour development: friend or foe? Nat Rev Cancer (2004) 4(12):966–77 10.1038/nrc1505 15573118

[184] Dong DZ Ni M Li JZ Xiong SG Ye W Virrey JJ Critical role of the stress chaperone GRP78/BiP in tumor proliferation, survival, and tumor angiogenesis in transgene-induced mammary tumor development. Cancer Res (2008) 68(2):498–505 10.1158/0008-5472.CAN-07-2950 18199545

[185] Reddy RK Mao CH Baumeister P Austin RC Kaufman RJ Lee AS Endoplasmic reticulum chaperone protein GRP78 protects cells from apoptosis induced by topoisomerase inhibitors – role of ATP binding site in suppression of caspase-7 activation. J Biol Chem (2003) 278(23):20915–24 10.1074/jbc.M212328200 12665508

[186] Sun S Lee D Ho AS Pu JK Zhang XQ Lee NP Inhibition of prolyl 4-hydroxylase, beta polypeptide (P4HB) attenuates temozolomide resistance in malignant glioma via the endoplasmic reticulum stress response (ERSR) pathways. Neuro Oncol (2013) 15(5):562–77 10.1093/neuonc/not005 23444257 PMC3635523

[187] Yamada M Tomida A Yun JS Cai B Yoshikawa H Taketani Y Cellular sensitization to cisplatin and carboplatin with decreased removal of platinum-DNA adduct by glucose-regulated stress. Cancer Chemother Pharmacol (1999) 44(1):59–64 10.1007/s002800050945 10367750

[188] Chatterjee S Hirota H Belfi CA Berger SJ Berger NA Hypersensitivity to DNA cross-linking agents associated with up-regulation of glucose-regulated stress protein GRP78. Cancer Res (1997) 57(22):5112–6 9371511

[189] Luvsandagva B Nakamura K Kitahara Y Aoki H Murata T Ikeda S GRP78 induced by estrogen plays a role in the chemosensitivity of endometrial cancer. Gynecol Oncol (2012) 126(1):132–9 10.1016/j.ygyno.2012.04.025 22543280

[190] Li JZ Lee AS Stress induction of GRP78/BiP and its role in cancer. Curr Mol Med (2006) 6(1):45–54 10.2174/156652406775574523 16472112

[191] Liu JF Fong YC Chang KW Kuo SC Chang CS Tang CH FPTB, a novel CA-4 derivative, induces cell apoptosis of human chondrosarcoma cells through mitochondrial dysfunction and endoplasmic reticulum stress pathways. J Cell Biochem (2011) 112(2):453–62 10.1002/jcb.22927 21268067

[192] Bett JS Ibrahim AF Garg AK Kelly V Pedrioli P Rocha S The P-body component USP52/PAN2 is a novel regulator of HIF1A mRNA stability. Biochem J (2013) 451:185–94 10.1042/BJ20130026 23398456 PMC3632086

[193] Chernov KG Barbet A Hamon L Ovchinnikov LP Curmi PA Pastre D Role of microtubules in stress granule assembly-microtubule dynamical instability favors the formation of micrometric stress granules in cells. J Biol Chem (2009) 284(52):36569–80 10.1074/jbc.M109.042879 19843517 PMC2794772

[194] Anderson P Kedersha N RNA granules. J Cell Biol (2006) 172(6):803–8 10.1083/jcb.200512082 16520386 PMC2063724

[195] Sriburi R Jackowski S Mori K Brewer JW XBP1: a link between the unfolded protein response, lipid biosynthesis, and biogenesis of the endoplasmic reticulum. J Cell Biol (2004) 167(1):35–41 10.1083/jcb.200406136 15466483 PMC2172532

[196] Schuck S Prinz WA Thorn KS Voss C Walter P Membrane expansion alleviates endoplasmic reticulum stress independently of the unfolded protein response. J Cell Biol (2009) 187(4):525–36 10.1083/jcb.200907074 19948500 PMC2779237

[197] Goyal U Blackstone C Untangling the web: mechanisms underlying ER network formation. Biochim Biophys Acta (2013) 1833(11):2492–8 10.1016/j.bbamcr.2013.04.009 23602970 PMC3729797

[198] Waterman-Storer CM Salmon ED Microtubule dynamics: treadmilling comes around again. Curr Biol (1997) 7(6):R369–72 10.1016/S0960-9822(06)00177-1 9197225

[199] Lee C Chen LB Dynamic behavior of endoplasmic-reticulum in living cells. Cell (1988) 54(1):37–46 10.1016/0092-8674(88)90177-8 3383243

[200] Ogburn KD Figueiredo-Pereira ME Cytoskeleton/endoplasmic reticulum collapse induced by prostaglandin J2 parallels centrosomal deposition of ubiquitinated protein aggregates. J Biol Chem (2006) 281(32):23274–84 10.1074/jbc.M600635200 16774923

[201] Wettstein G Bellaye PS Micheau O Bonniaud P Small heat shock proteins and the cytoskeleton: an essential interplay for cell integrity? Int J Biochem Cell Biol (2012) 44(10):1680–6 10.1016/j.biocel.2012.05.024 22683760

[202] Ghosh JG Houck SA Clark JI Interactive domains in the molecular chaperone human alpha B crystallin modulate microtubule assembly and disassembly. PLoS One (2007) 2(6):e498 10.1371/journal.pone.0000498 17551579 PMC1876262

[203] Houck SA Clark JI Dynamic subunit exchange and the regulation of microtubule assembly by the stress response protein human alpha B crystallin. PLoS One (2010) 5(7):e11795 10.1371/journal.pone.0011795 20668689 PMC2909917

[204] Fujita Y Ohto E Katayama E Atomi Y Alpha B-crystallin-coated MAP microtubule resists nocodazole and calcium-induced disassembly. J Cell Sci (2004) 117(9):1719–26 10.1242/jcs.01021 15075233

[205] Hino M Kurogi K Okubo MA Murata-Hori M Hosoya H Small heat shock protein 27 (HSP27) associates with tubulin/microtubules in HeLa cells. Biochem Biophys Res Commun (2000) 271(1):164–9 10.1006/bbrc.2000.2553 10777697

[206] Almeida-Souza L Asselbergh B De Winter V Goethals S Timmerman V Janssens S HSPB1 facilitates the formation of non-centrosomal microtubules. PLoS One (2013) 8(6):e66541 10.1371/journal.pone.0066541 23826100 PMC3691211

[207] Casado P Zuazua-Villar P Prado MA del Valle E Iglesias JM Martinez-Campa C Characterization of HSP27 phosphorylation induced by microtubule interfering agents: implication of p38 signalling pathway. Arch Biochem Biophys (2007) 461(1):123–9 10.1016/j.abb.2007.01.027 17367746

[208] Sanchez C Padilla R Paciucci R Zabala JC Avila J Binding of heat-shock protein-70 (hsp70) to tubulin. Arch Biochem Biophys (1994) 310(2):428–32 10.1006/abbi.1994.1188 8179328

[209] Welch WJ Feramisco JR Blose SH The mammalian stress response and the cytoskeleton – alterations in intermediate filaments. Ann N Y Acad Sci (1985) 455:57–67 10.1111/j.1749-6632.1985.tb50403.x 3866510

[210] Selimovic D Badura HE El-Khattouti A Soell M Porzig B Spernger A Vinblastine-induced apoptosis of melanoma cells is mediated by Ras homologous A protein (Rho A) via mitochondrial and non-mitochondrial-dependent mechanisms. Apoptosis (2013) 18(8):980–97 10.1007/s10495-013-0844-4 23564313

[211] Guo SH Wharton W Moseley P Shi HL Heat shock protein 70 regulates cellular redox status by modulating glutathione-related enzyme activities. Cell Stress Chaperones (2007) 12(3):245–54 10.1379/CSC-265.1 17915557 PMC1971240

[212] Quintá HR Galigniana NM Erlejman AG Lagadari M Piwien-Pilipuk G Galigniana MD Management of cytoskeleton architecture by molecular chaperones and immunophilins. Cell Signal (2011) 23(12):1907–20 10.1016/j.cellsig.2011.07.023 21864675 PMC3184352

[213] Hong DS Banerji U Tavana B George GC Aaron J Kurzrock R Targeting the molecular chaperone heat shock protein 90 (HSP90): lessons learned and future directions. Cancer Treat Rev (2013) 39(4):375–87 10.1016/j.ctrv.2012.10.001 23199899

[214] Weis F Moullintraffort L Heichette C Chretien D Garnier C The 90-kDa heat Shock protein Hsp90 protects tubulin against thermal denaturation. J Biol Chem (2010) 285(13):9525–34 10.1074/jbc.M109.096586 20110359 PMC2843203

[215] Pratt WB Toft DO Regulation of signaling protein function and trafficking by the hsp90/hsp70-based chaperone machinery. Exp Biol Med (2003) 228(2):111–33 12563018 10.1177/153537020322800201

[216] Pratt WB Morishima Y Peng HM Osawa Y Proposal for a role of the Hsp90/Hsp70-based chaperone machinery in making triage decisions when proteins undergo oxidative and toxic damage. Exp Biol Med (2010) 235(3):278–89 10.1258/ebm.2009.009250 20404045 PMC3046050

[217] Rovini A Savry A Braguer D Carre M Microtubule-targeted agents: when mitochondria become essential to chemotherapy. Biochim Biophys Acta (2011) 1807(6):679–88 10.1016/j.bbabio.2011.01.001 21216222

[218] Ren Y Zhao JH Feng J Parkin binds to alpha/beta tubulin and increases their ubiquitination and degradation. J Neurosci (2003) 23(8):3316–24 12716939 10.1523/JNEUROSCI.23-08-03316.2003 PMC1876717

[219] Jung D Filliol D Miehe M Rendon A Interaction of brain mitochondria with microtubules reconstituted from brain tubulin and MAP2 or Tau. Cell Motil Cytoskeleton (1993) 24(4):245–55 10.1002/cm.970240405 8097434

[220] Maldonado EN Patnaik J Mullins MR Lemasters JJ Free tubulin modulates mitochondrial membrane potential in cancer cells. Cancer Res (2010) 70(24):10192–201 10.1158/0008-5472.CAN-10-2429 21159641 PMC3010233

[221] Guzun R Karu-Varikmaa M Gonzalez-Granillo M Kuznetsov AV Michel L Cottet-Rousselle C Mitochondria-cytoskeleton interaction: distribution of beta-tubulins in cardiomyocytes and HL-1 cells. Biochim Biophys Acta (2011) 1807(4):458–69 10.1016/j.bbabio.2011.01.010 21296049

[222] Andre N Braguer D Brasseur G Goncalves A Lemesle-Meunier D Guise S Paclitaxel induces release of cytochrome c from mitochondria isolated from human neuroblastoma cells. Cancer Res (2000) 60(19):5349–53 11034069

[223] Ferlini C Raspaglio G Mozzetti S Distefano M Filippetti F Martinelli E Bcl-2 down-regulation is a novel mechanism of paclitaxel resistance. Mol Pharmacol (2003) 64(1):51–8 10.1124/mol.64.1.51 12815160

[224] Gajate C Barasoain I Andreu JM Mollinedo F Induction of apoptosis in leukemic cells by the reversible microtubule-disrupting agent 2-methoxy-5-(2’,3’,4’-trimethoxyphenyl)-2,4,6-cycloheptatrien-1-one: protection by bcl-2 and bcl-x(L) and cell cycle arrest. Cancer Res (2000) 60(10):2651–9 10825137

[225] Ibrado AM Liu L Bhalla K Bcl-x(L) overexpression inhibits progression of molecular events leading to paclitaxel-induced apoptosis of human acute myeloid leukemia HL-60 cells. Cancer Res (1997) 57(6):1109–15 9067280

[226] Esteve MA Carre M Bourgarel-Rey V Kruczynski A Raspaglio G Ferlini C Bcl-2 down-regulation and tubulin subtype composition are involved in resistance of ovarian cancer cells to vinflunine. Mol Cancer Ther (2006) 5(11):2824–33 10.1158/1535-7163.MCT-06-0277 17121929

[227] Knipling L Wolff J Direct interaction of Bcl-2 proteins with tubulin. Biochem Biophys Res Commun (2006) 341(2):433–9 10.1016/j.bbrc.2005.12.201 16446153

[228] Puthalakath H Huang DC O’Reilly LA King SM Strasser A The proapoptotic activity of the Bcl-2 family member Bim is regulated by interaction with the dynein motor complex. Mol Cell (1999) 3(3):287–96 10.1016/S1097-2765(00)80456-6 10198631

[229] Giodini A Kallio MJ Wall NR Gorbsky GJ Tognin S Marchisio PC Regulation of microtubule stability and mitotic progression by survivin. Cancer Res (2002) 62(9):2462–7 11980633

[230] Prislei S Mozzetti S Filippetti F De Donato M Raspaglio G Cicchillitti L From plasma membrane to cytoskeleton: a novel function for semaphorin 6A. Mol Cancer Ther (2008) 7(1):233–41 10.1158/1535-7163.MCT-07-0390 18187809

[231] Srivastava RK Srivastava AR Korsmeyer SJ Nesterova M Cho-Chung YS Longo DL Involvement of microtubules in the regulation of Bcl2 phosphorylation and apoptosis through cyclic AMP-dependent protein kinase. Mol Cell Biol (1998) 18(6):3509–17 9584191 10.1128/mcb.18.6.3509 PMC108932

[232] Ling YH Tornos C Perez-Soler R Phosphorylation of Bcl-2 is a marker of M phase events and not a determinant of apoptosis. J Biol Chem (1998) 273(30):18984–91 10.1074/jbc.273.30.18984 9668078

[233] Verdier-Pinard P Pasquier E Xiao H Burd B Villard C Lafitte D Tubulin proteomics: towards breaking the code. Anal Biochem (2009) 384(2):197–206 10.1016/j.ab.2008.09.020 18840397 PMC4039029

[234] Hasegawa S Miyoshi Y Egawa C Ishitobi M Taguchi T Tamaki Y Prediction of response to docetaxel by quantitative analysis of class I and III beta-tubulin isotype mRNA expression in human breast cancers. Clin Cancer Res (2003) 9(8):2992–7 12912947

[235] Zhang HL Ruan L Zheng LM Whyte D Tzeng CM Zhou XW Association between class III beta-tubulin expression and response to paclitaxel/vinorebine-based chemotherapy for non-small cell lung cancer: a meta-analysis. Lung Cancer (2012) 77(1):9–15 10.1016/j.lungcan.2012.01.005 22306125

[236] Sève P Lai R Ding K Winton T Butts C Mackey J Class III β-tubulin expression and benefit from adjuvant cisplatin/vinorelbine chemotherapy in operable non-small cell lung cancer: analysis of NCIC JBR.10. Clin Cancer Res (2007) 13(3):994–9 10.1158/1078-0432.CCR-06-1503 17289895

[237] Reiman T Lai R Veillard AS Paris E Soria JC Rosell R Cross-validation study of class III beta-tubulin as a predictive marker for benefit from adjuvant chemotherapy in resected non-small-cell lung cancer: analysis of four randomized trials. Ann Oncol (2012) 23(1):86–U10 10.1093/annonc/mdr033 21471564 PMC3276322

[238] Seve P Isaac S Tredan O Souquet PJ Pacheco Y Perol M Expression of class III beta-tubulin is predictive of patient outcome in patients with non-small cell lung cancer receiving vinorelbine-based chemotherapy. Clin Cancer Res (2005) 11(15):5481–6 10.1158/1078-0432.CCR-05-0285 16061864

[239] Ohishi Y Oda Y Basaki Y Kobayashi H Wake N Kuwano M Expression of beta-tubulin isotypes in human primary ovarian carcinoma. Gynecol Oncol (2007) 105(3):586–92 10.1016/j.ygyno.2007.01.044 17343904

[240] Kavallaris M Kuo DY Burkhart CA Regl DL Norris MD Haber M Taxol-resistant epithelial ovarian tumors are associated with altered expression of specific beta-tubulin isotypes. J Clin Invest (1997) 100(5):1282–93 10.1172/JCI119642 9276747 PMC508306

[241] De Donato M Mariani M Petrella L Martinelli E Zannoni GF Vellone V Class III beta-tubulin and the cytoskeletal gateway for drug resistance in ovarian cancer. J Cell Physiol (2012) 227(3):1034–41 10.1002/jcp.22813 21520077

[242] Su D Smith SM Preti M Schwartz P Rutherford TJ Menato G Stathmin and tubulin expression and survival of ovarian cancer patients receiving platinum treatment with and without paclitaxel. Cancer (2009) 115(11):2453–63 10.1002/cncr.24282 19322891

[243] Aoki D Oda Y Hattori S Taguchi K Ohishi Y Basaki Y Overexpression of class III beta-tubulin predicts good response to taxane-based chemotherapy in ovarian clear cell adenocarcinoma. Clin Cancer Res (2009) 15(4):1473–80 10.1158/1078-0432.CCR-08-1274 19228748

[244] Paradiso A Mangia A Chiriatti A Tommasi S Zito A Latorre A Biomarkers predictive for clinical efficacy of taxol-based chemotherapy in advanced breast cancer. Ann Oncol (2005) 16:14–9 10.1093/annonc/mdi902 15923415

[245] Portyanko A Kovalev P Gorgun J Cherstvoy E Beta(III)-tubulin at the invasive margin of colorectal cancer: possible link to invasion. Virchows Arch (2009) 454(5):541–8 10.1007/s00428-009-0764-4 19360438

[246] Roque DM Bellone S English DP Buza N Cocco E Gasparrini S Tubulin-beta-III overexpression by uterine serous carcinomas is a marker for poor overall survival after platinum/taxane chemotherapy and sensitivity to epothilones. Cancer (2013) 119(14):2582–92 10.1002/cncr.28017 23585021 PMC3700638

[247] Hwang JE Hong JY Kim K Kim SH Choi WY Kim MJ Class III beta-tubulin is a predictive marker for taxane-based chemotherapy in recurrent and metastatic gastric cancer. BMC Cancer (2013) 13:431 10.1186/1471-2407-13-431 24053422 PMC4015872

[248] Ploussard G Terry S Maille P Allory Y Sirab N Kheuang L Class III beta-tubulin expression predicts prostate tumor aggressiveness and patient response to docetaxel-based chemotherapy. Cancer Res (2010) 70(22):9253–64 10.1158/0008-5472.CAN-10-1447 21045157 PMC3290526

[249] Terry S Ploussard G Allory Y Nicolaiew N Boissiere-Michot F Maille P Increased expression of class III beta-tubulin in castration-resistant human prostate cancer. Br J Cancer (2009) 101(6):951–6 10.1038/sj.bjc.6605245 19690549 PMC2743364

[250] Bernard-Marty C Treilleux I Dumontet C Cardoso F Fellous A Gancberg D Microtubule-associated parameters as predictive markers of docetaxel activity in advanced breast cancer patients: results of a pilot study. Clin Breast Cancer (2002) 3(5):341–5 10.3816/CBC.2002.n.037 12533264

[251] Christoph DC Kasper S Gauler TC Loesch C Engelhard M Theegarten D BetaV-tubulin expression is associated with outcome following taxane-based chemotherapy in non-small cell lung cancer. Br J Cancer (2012) 107(5):823–30 10.1038/bjc.2012.324 22836512 PMC3425975

[252] Lu CH Zhang J He S Wan CH Shan AD Wang YY Increased alpha-tubulin1b expression indicates poor prognosis and resistance to chemotherapy in hepatocellular carcinoma. Dig Dis Sci (2013) 58(9):2713–20 10.1007/s10620-013-2692-z 23625295

[253] Caracciolo V D’Agostino L Draberova E Sladkova V Crozier-Fitzgerald C Agamanolis DP Differential expression and cellular distribution of gamma-tubulin and beta III-tubulin in medulloblastomas and human medulloblastoma cell lines. J Cell Physiol (2010) 223(2):519–29 10.1002/jcp.22077 20162618

